# Occupational Stressors, Working Conditions and Self-Reported Mental Health Among Veterinarians in Germany: An Exploratory Cross-Sectional Survey

**DOI:** 10.3390/healthcare14172749

**Published:** 2026-08-28

**Authors:** Marietta Máté, Anneke Jensch, László Ózsvári

**Affiliations:** Department of Veterinary Forensics and Economics, University of Veterinary Medicine Budapest, István Street 2, 1078 Budapest, Hungary; anneke_jensch@web.de (A.J.); ozsvari.laszlo@univet.hu (L.Ó.)

**Keywords:** veterinarians, occupational stress, mental health, job satisfaction, work–life balance, burnout, working conditions, Germany

## Abstract

**Highlights:**

**What are the main findings?**

- Work–life balance and working hours were the highest-rated occupational stressors among veterinarians working in Germany.
- Stress-related symptoms were common, particularly sleep disturbances, burnout symptoms and anxiety, while workplace psychological support was rarely available or used.

**What are the implications of the main findings?**

- Preventive strategies should address not only individual coping strategies but also working-time arrangements, staffing, administrative workload, and workplace support.
- Veterinary workplaces and professional organizations should provide accessible psychological support and promote working environments that support long-term job satisfaction and professional sustainability.

**Abstract:**

**Background/Objectives:** Veterinarians are exposed to substantial psychological strain, including burnout, depressive symptoms, and increased suicide risk. Emotional and ethical challenges, such as managing animal suffering, making difficult treatment decisions, and handling conflicts with animal owners, may compound the pressures associated with demanding working conditions. This study aimed to investigate occupational stressors, working conditions, job satisfaction, and self-reported mental health among veterinarians working in Germany. **Methods:** An exploratory, quantitative, cross-sectional online survey was conducted among veterinarians working in Germany between August and October 2025. A total of 126 veterinarians completed the questionnaire. Associations between demographic and professional characteristics, working conditions, occupational stressors, and self-reported mental health indicators were analyzed using Pearson’s chi-square tests, Mann–Whitney U tests and Kruskal–Wallis tests. **Results:** The greatest perceived occupational stress was associated with work–life balance and working hours (mean ± SD: 3.67 ± 1.14), followed by the management of complex medical cases and communication with animal owners. Younger veterinary respondents reported greater stress related to selection of treatment options, whereas older and more experienced veterinary respondents reported greater stress related to administrative workload. Women reported higher stress levels across several domains, including communication with animal owners and the management of difficult medical cases. Veterinarians working more than 40 h per week reported lower satisfaction with work–life balance, while those with more than 28 days of annual leave reported greater workplace comfort. Self-reported stress-related symptoms were frequently indicated in the symptom checklist: 63.5% reported sleep disturbances, 31.7% burnout symptoms, and 26.2% anxiety. Only 19.0% had sought psychological help for work-related stress, and workplace psychological support was available and used by only 3.2% of respondents. **Conclusions:** The exploratory findings indicate substantial perceived work-related, emotional, and ethical burdens among veterinarians working in Germany. Improved working-time arrangements, administrative support, accessible psychological support, and broader organizational measures may help protect veterinarians’ well-being and long-term job satisfaction.

## 1. Introduction

Veterinary medicine is widely recognized as a psychologically demanding profession that requires not only advanced clinical knowledge and decision-making skills, but also substantial emotional resilience. Veterinarians are regularly exposed to a range of occupational stressors, including high workload, long and irregular working hours, night and weekend duties, emergency care, emotionally demanding interactions with animal owners, financial constraints affecting treatment decisions, and ethical dilemmas related to euthanasia and animal welfare [1,2,3]. Although veterinary work is often perceived by the public as both respected and meaningful, its psychological burden has become an increasingly important topic of international research [1,2].

Evidence from several countries indicates that veterinarians are at increased risk of work-related stress, burnout, psychological distress, depression, and suicidal ideation compared with the general population and, in some studies, with other occupational groups [1,2,3]. A study from the United Kingdom showed that psychosocial working conditions, long working hours, client-related stressors and work–life conflict are strongly associated with poor mental well-being among veterinary surgeons [1]. Research from the United States has similarly highlighted the role of client expectations, educational debt, long working hours, ethical conflicts, and access to lethal means in veterinarians’ mental health risks [4,5]. Canadian and Australian studies have also reported high levels of burnout, compassion fatigue, anxiety, and depressive symptoms among veterinary professionals, indicating that these challenges are not limited to a single national context [6,7].

In Europe, the available evidence suggests both shared and country-specific patterns. German-speaking and Central European studies have identified time pressure, overtime, on-call duties, demanding clients, administrative workload, and difficulties in reconciling work and private life as major stressors [2,8,9,10]. In Austria, communication with animal owners, night and weekend duties, and financial and ethical conflicts have been described as important burdens [9]. Recent Slovenian data showed that work–life imbalance, ethical conflicts, and long working hours were significant predictors of burnout symptoms, with younger and female veterinarians being particularly affected [11]. A Croatian cross-sectional survey also emphasized the importance of assessing work-related stressors, career satisfaction, and mental health within national veterinary contexts, as such data may help identify country-specific needs and preventive strategies [12]. More recently, Spanish data indicated that emotional exhaustion and affective symptoms are closely linked to suicidal ideation among veterinary professionals, further underlining the importance of emotional strain as a clinically relevant component of occupational stress [13].

A large European survey comparing data from 2018/2019 and 2022/2023 also reported persistently high stress levels and reduced mental well-being among veterinarians, with early-career and female veterinarians being particularly affected [14]. Previous cross-national European findings also support the importance of examining stressors across different professional and national contexts. Máté et al. [15] reported substantial mental health concerns and suicidal ideation among veterinarians from Hungary, Finland, Sweden, Germany, and other Northern European countries, with younger veterinarians more often reporting negative mental health impacts and female veterinarians more frequently seeking professional counselling. In a related analysis of the same survey, Máté et al. [16] found that fatigue, emotional exhaustion, burnout-related symptoms, client-related stressors, complaints, and negative online comments were among the most important perceived burdens affecting both veterinary work and personal life.

Germany is a particularly relevant setting for further investigation. Previous German studies have reported higher levels of depressive symptoms and suicidal ideation among veterinarians compared with the general population [2,8]. Studies of work-related behavior and experience patterns have shown that a substantial proportion of German veterinarians exhibit health-risk patterns, especially those characterized by overcommitment and burnout-related tendencies [8,10]. Younger veterinarians appear to be especially vulnerable to overcommitment, cognitive and emotional irritation, and reduced work and life satisfaction [10]. Recent German findings also suggest that cognitive and emotional irritation are associated with work overload, limited recovery, and declining well-being, highlighting the need to better understand modifiable workplace stressors in this professional group [10].

Veterinary work in Germany is shaped by several structural and organizational factors that may influence perceived stress. Veterinarians frequently face long working hours, on-call and weekend duties, administrative requirements, client pressure, and economic constraints. At the same time, the profession has undergone demographic and structural changes, including feminization, changing career expectations, and increasing emphasis on work–life balance [2,10,17]. In another previous survey, Máté et al. [18] showed that, among Hungarian and German veterinarians, a positive work atmosphere, work–life balance, and predictable working hours were among the most important motivational factors, while income expectations and professional autonomy differed between countries.

Occupational stress may affect veterinarians at several levels. At the individual level, chronic stress has been associated with sleep disturbances, emotional exhaustion, anxiety, depressive symptoms, compassion fatigue, and lower job satisfaction [2,3,8,9,19]. At the professional level, stress may impair concentration, decision-making, client communication, and perceived work performance [3,15,19]. At the organizational level, prolonged strain may contribute to staff turnover, intentions to leave the profession, presenteeism, and reduced sustainability of veterinary services [3,15,18]. Recent research also suggests that occupational stress does not necessarily directly lead to severe mental health outcomes. For example, Gresele et al. [20], in a large Brazilian study, identified psychological distress as a central mediating mechanism linking occupational stress, compassion fatigue, the workplace environment, and coping strategies to suicidal ideation. This supports the view that workplace stressors, emotional processes, and coping resources should be examined together rather than as isolated factors.

Demographic and occupational characteristics may further shape how stressors are perceived and experienced. Previous studies indicate that younger and early-career veterinarians are more likely to report emotional exhaustion, uncertainty in clinical decision-making, difficulties in client communication, and greater psychological strain [2,3,9,10,15,19]. Gender differences have also been reported, with female veterinarians more frequently reporting anxiety, emotional exhaustion, and difficulties maintaining work–life balance in several national studies [1,2,3,21]. Working hours, professional position, years of experience, field of practice, number of annual leave days, and organizational support may also influence perceived stress, job satisfaction, and mental health outcomes [4,10,15,22,23].

Despite the growing international literature, there remains a need for context-specific evidence from Germany examining the relationships between everyday occupational stressors, professional decision-making, job satisfaction, and mental health. Existing German studies have provided important insights into overcommitment, irritation, psychosocial strain, and work-related behavioral patterns [8,10,24], but less is known about how a broader range of specific stressors is perceived in daily veterinary work and whether these perceptions differ according to demographic and occupational characteristics. Such information is necessary for developing targeted, evidence-based preventive measures that address not only individual coping, but also modifiable organizational conditions.

Therefore, the present study aimed to provide an exploratory assessment of work-related stressors, job satisfaction and self-reported mental health among veterinarians working in Germany. The specific objectives were: (1) to identify the most relevant perceived stressors in daily veterinary work; (2) to examine veterinarians’ perceptions of how these stressors relate to professional decision-making, and perceived work performance; (3) to assess self-reported mental health, well-being, and job satisfaction outcomes; and (4) to explore whether perceived stressors and their reported professional and psychological consequences differed according to demographic and occupational characteristics, including age, gender, professional position, weekly working hours, number of annual leave days, and years of experience, as well as self-reported mental health status.

## 2. Materials and Methods

### 2.1. Research Design and Questionnaire Development

The present study employed an exploratory quantitative cross-sectional design to investigate work-related stressors, job satisfaction, and self-reported mental health among veterinarians practicing in Germany. An online questionnaire was chosen to ensure broad geographic coverage, anonymity, and efficient data collection among veterinarians.

The questionnaire was developed following a comprehensive review of the literature on occupational stress, psychosocial demands, mental health, and job satisfaction in the veterinary profession. The theoretical background was informed by two established frameworks in occupational stress research: the Transactional Model of Stress and Coping [25] and the Job Demand–Control Model [26]. These models guided the thematic structure of the questionnaire but were not directly operationalized as measurement instruments. During the preparatory phase, validated instruments, including the Copenhagen Burnout Inventory and the Job Content Questionnaire, were reviewed [2,19,24,26,27,28,29]. However, the final survey consisted of original, study-specific items developed for the veterinary context and aligned with the aims of the present exploratory study [1,3,9].

Based on the literature review, an initial item pool was developed to cover key occupational stressors and psychosocial demands in veterinary work, including workload, emotional strain, communication challenges, administrative burden, financial considerations, ethical dilemmas, work–life balance, job satisfaction, and workplace support. The item pool was subsequently refined through discussions among members of the research team. The original questionnaire was developed in English and then translated into German. The translation process involved consultation within the research team, including one co-author with advance bilingual proficiency in both English and German. Any inconsistencies or ambiguities were discussed and resolved by consensus to ensure conceptual and linguistic equivalence between the two versions.

Before finalization, a pilot version of the questionnaire was tested with a small group of veterinarians (*n* = 10) to assess its clarity, comprehensibility, technical functionality, and thematic completeness. Feedback from the pilot phase led to minor wording adjustments and the refinement of response options. No formal psychometric validation of the questionnaire was performed. The pilot testing was also intended to support conceptual consistency between the English and German versions. The final questionnaire (Supplementary Questionnaire) consisted of 43 items grouped into five sections:

- Demographic and occupational characteristics (Q1–Q11): age, gender, federal state, field of activity, employment status, weekly working hours, annual leave entitlement, professional experience, on-call duties, and availability of support staff.
- General mental health and stress-related symptoms (Q12–Q18): current mental health status, occurrence of stress-related symptoms (e.g., sleep disturbances, anxiety, burnout symptoms), prior psychological help-seeking, perceived effects of animal suffering, rumination after work, recovery during annual leave, and availability of workplace psychological support.
- Work-related stressor items (Likert scale) (Q19–Q36): communication demands, medical decision-making, administrative tasks, financial considerations, workload, and professional development challenges.
- Job satisfaction and psychosocial work environment (Q37–Q42): satisfaction with work–life balance, sense of belonging within the team, communication with colleagues and supervisors, workplace attitudes toward mental health, and overall job satisfaction.
- Open-ended comments (Q43): an optional field for reporting additional stressors or remarks not covered by closed-ended items.

All Likert-scale items used a five-point response format, where 1 = strongly disagree, 2 = disagree, 3 = neither agree nor disagree, 4 = agree, and 5 = strongly agree. The items were analyzed individually, and internal consistency was assessed for relevant multi-item sets.

### 2.2. Data Collection and Sample Description

Data collection was conducted from 20 August to 31 October 2025 using Google Forms. A convenience sampling strategy was applied because of the exploratory nature of the study. No a priori sample size calculation was performed; instead, the aim was to recruit as large and diverse a sample as possible within the survey period. A link to the questionnaire was distributed together with a short introductory text inviting veterinarians to participate. The introductory text outlined the purpose of the study, summarized the thematic focus of the questionnaire, and informed participants about the expected completion time, and explained that responses would be collected anonymously and how the data would subsequently be processed and analyzed.

The questionnaire was disseminated through several digital channels to reach veterinarians across Germany. It was posted in a Facebook group for veterinarians, and circulated through personal contacts within the veterinary community. This multi-channel recruitment strategy was intended to reach veterinarians from different age groups, fields of veterinary activity, employment statuses, and geographical regions. Participation was voluntary and anonymous. Before completing the questionnaire, all respondents provided written informed consent electronically. All study procedures were approved by the Scientific and Innovation Committee of the University of Veterinary Medicine Budapest.

Eligibility criteria required respondents to hold a veterinary degree and to be professionally active in the veterinary field in Germany during the data collection period. Before analysis, responses were checked for completeness, eligibility, and logical consistency. A total of 18 responses were excluded during data cleaning because they consisted of incomplete questionnaires or were submitted by individuals who did not meet the eligibility criteria, including retired veterinarians or veterinarians not professionally active in Germany during the study period. This resulted in a final analytical sample of 126 valid and complete responses. As the questionnaire was fully anonymous, respondents could not be individually identified. Data were exported from Google Forms and prepared for data cleaning and descriptive analysis in Microsoft Excel, Version 2608 (Microsoft Corporation, Redmond, WA, USA).

### 2.3. Statistical Analysis

Data were analyzed using IBM SPSS Statistics, version 25 (IBM Corp., Armonk, NY, USA) [30]. The individual veterinarian was the unit of analysis. The questionnaire included five-point Likert-scale items, where 1 = strongly disagree, 2 = disagree, 3 = neither agree nor disagree, 4 agree, and 5 strongly agree. Although Likert-scale responses are ordinal by design, descriptive statistics, including means and standard deviations (mean ± SD), were calculated for each item to summarize response patterns and facilitate interpretation and comparison between groups. The normality of the distributions of the Likert-scale items was assessed using the Shapiro–Wilk test. As all variables showed significant deviations from normality (Shapiro–Wilk test, *p* < 0.05), non-parametric tests were used for all subsequent group comparisons involving Likert-scale items.

Associations between categorical variables were examined using Pearson’s chi-square (χ^2^) tests. These analyses assessed relationships between demographic and occupational characteristics, including age group, gender, professional position, weekly working hours, number of annual leave days, years of professional experience and perceived mental health status, and categorical questionnaire outcomes. For each categorical comparison, cross-tabulations were created, and the results of Pearson’s chi-square tests were reported with the χ^2^ statistic, degrees of freedom (df) and *p*-value. Cramer’s V was calculated as a measure of effect size for the chi-square tests. Values below 0.10 were interpreted as indicating negligible or weak associations, values between 0.10 and 0.30 as indicating small-to-moderate associations, and values above 0.30 as indicating moderate or stronger associations. These thresholds were interpreted cautiously because effect-size interpretation may depend on the dimensions of the contingency table, as well as on the exploratory design and the use of convenience sampling [31].

For Likert-type outcomes, two complementary analytical approaches were reported. Mann–Whitney *U* tests and Kruskal–Wallis tests were considered the primary analyses for group comparisons because the five-point Likert responses were ordinal and non-normally distributed. Pearson’s chi-square tests were retained as supplementary analyses to examine whether the distribution of response categories differed between groups, with the five Likert response options treated as categorical response levels. Thus, the non-parametric tests assessed differences in ordinal item scores, whereas chi-square tests assessed differences in the overall distribution of response categories.

For comparisons between two independent groups, such as groups defined by gender, professional position, weekly working hours, and number of annual leave days, the Mann–Whitney *U* test was applied. Results were reported with *U* values, *Z* values, and two-tailed *p*-values. For comparisons involving three or more groups, such as age group, years of professional experience, and perceived mental health status, the Kruskal–Wallis test was used. Results were presented as *H* statistics, degrees of freedom, and *p*-values. Mean ranks were examined to determine the direction and the observed group differences. For statistically significant Kruskal–Wallis tests, Dunn–Bonferroni post hoc pairwise comparisons were performed to identify which groups differed. Bonferroni-adjusted *p*-values were used to guide the interpretation of pairwise group differences.

The internal consistency of predefined multi-item groups was assessed using Cronbach’s alpha (α). Cronbach’s alpha was calculated at the scale level and not for individual questionnaire items. The item groups reflected conceptually related domains of the questionnaire: daily work-related stressors, professional decision-making factors, and job satisfaction and workplace well-being. According to established criteria, α ≥ 0.70 was considered acceptable, α ≥ 0.80 was considered good, and α ≥ 0.90 was considered excellent [32]. In the present dataset, Cronbach’s alpha coefficients were 0.743 for daily work-related stressors, 0.794 for professional decision-making factors, and 0.862 for job satisfaction and workplace well-being, indicating acceptable to good internal consistency across the predefined item groups (Supplementary Table S1). Corrected item-total correlations and Cronbach’s alpha values if individual items were deleted were examined as diagnostic statistics. As the removal of individual items did not meaningfully improve internal consistency and no major conceptual inconsistencies were identified, all items were retained.

A two-sided significance level of *p* < 0.05 was used throughout. Because of the exploratory design and the large number of subgroup comparisons across questionnaire items, no formal adjustment for multiple testing was applied across all analyses. Therefore, *p*-values should be interpreted as nominal and hypothesis-generating rather than confirmatory. Isolated statistically significant findings were interpreted cautiously and primarily used to identify patterns for further investigation.

## 3. Results

### 3.1. Sociodemographic and Occupational Data of the Participants

**Figure 1:**
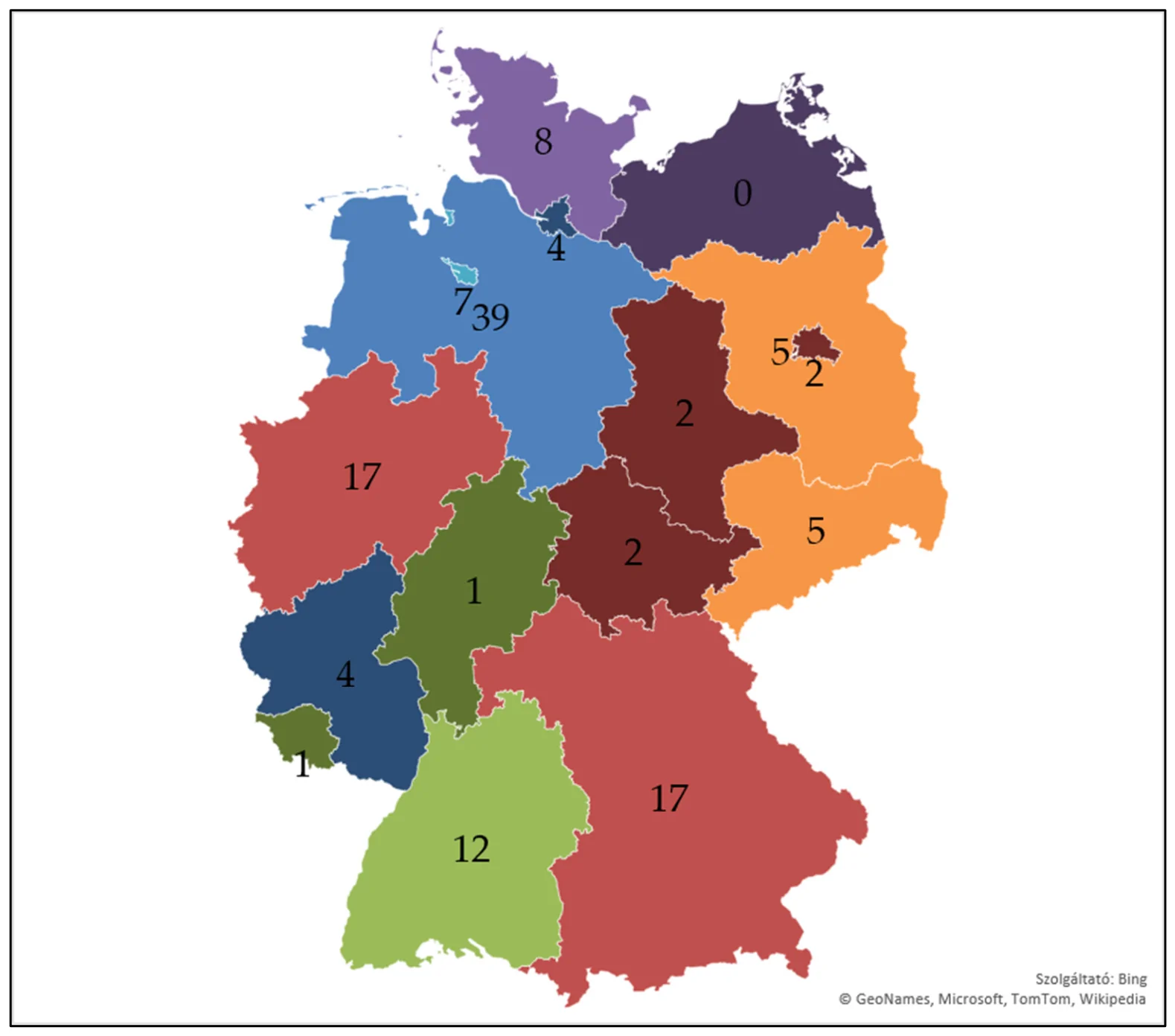
Geographical distribution of participants across the German federal states (*n* = 126). Map generated using Microsoft Bing Maps. Microsoft product screenshot reprinted with permission from Microsoft Corporation. The original attribution displayed on the map is retained.

A total of 126 veterinarians were included in the study. Participants represented 15 of Germany’s 16 federal states. The largest proportion of participants came from Lower Saxony (31.0%), followed by Bavaria (13.5%), North Rhine-Westphalia (13.5%), Baden-Württemberg (9.5%), and Schleswig-Holstein (6.3%). Mecklenburg-Vorpommern was the only federal state not represented in the sample (Figure 1).

Nearly half of all respondents were between 23 and 34 years of age (46.0%). The sample consisted of substantially more women (80.2%) than men (19.8%). Small animal practice was the most frequently reported field of veterinary activity (71.4%). The “Other” category comprised respondents working in veterinary-related industry and commercial roles (e.g., pharmaceutical sector, field service, or consultancy) as well as those working in practice management or advisory positions. Further demographic and occupational characteristics are summarized in Table 1. For comparison, the German Federal Chamber of Veterinarians (Bundestierärztekammer, BTK) reported that women accounted for 66.8% of all members of the German veterinary chambers and 71.7% of actively working veterinarians in Germany in 2025 [33]. However, official national statistics do not provide a directly comparable individual-level estimate of the proportion of veterinarians working in small animal practice.

### 3.2. Stress Factors in Daily Work

**Figure 2:**
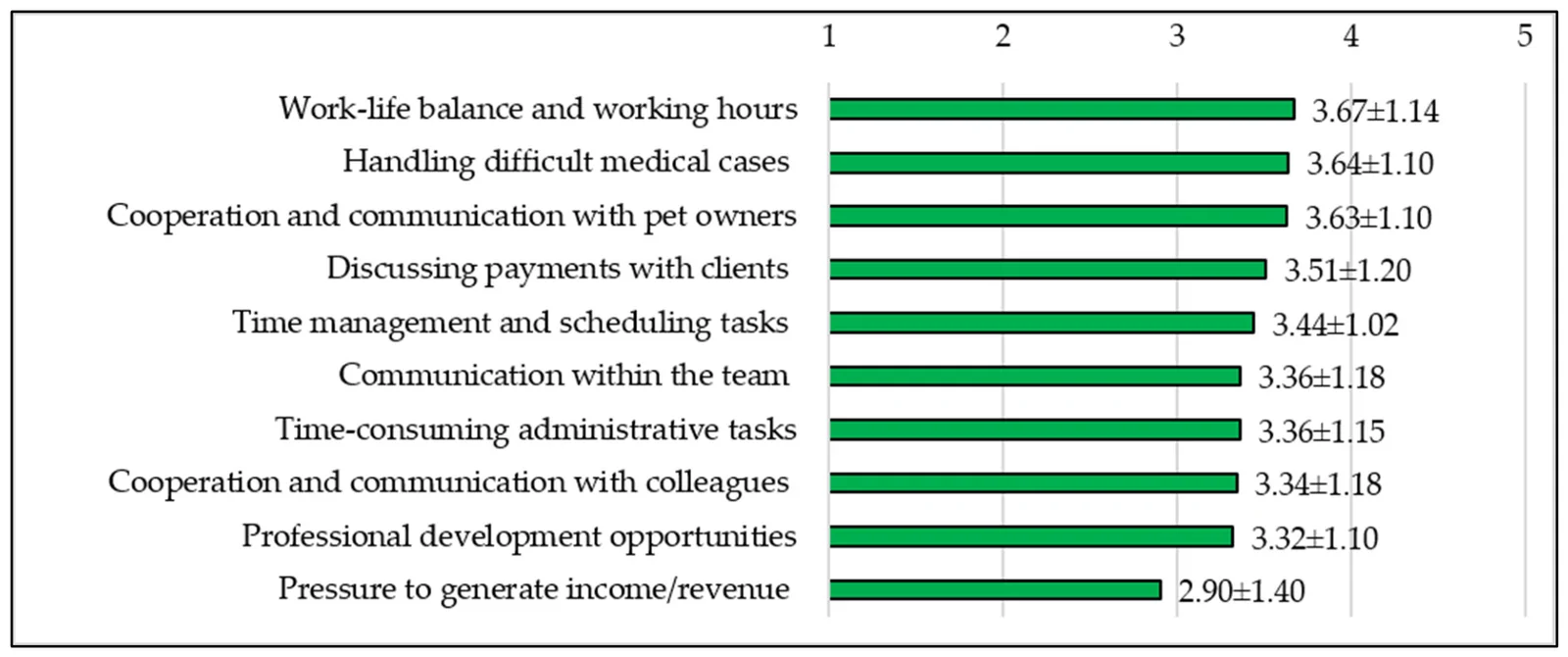
Veterinarians’ perceptions of work-related stressors in daily veterinary work (mean ± SD) (*n* = 126). Responses were given on a five-point Likert scale, where 1 = strongly disagree, 2 = disagree, 3 = neither agree nor disagree, 4 = agree, and 5 = strongly agree.

The analysis of daily work-related stressors showed that respondents reported moderate to relatively high levels of perceived stress across several areas (Figure 2). The highest mean score was observed for difficulties related to work–life balance and working hours (mean ± SD: 3.67 ± 1.14). Similarly high scores were observed for handling difficult medical cases and cooperating and communicating with animal owners. Administrative and interpersonal aspects also contributed to perceived stress. Time management, task scheduling, and team communication was reported as moderately stressful, while stress related to administrative work and cooperation with colleagues was reported at comparable levels. Lower mean scores were observed for limitations in professional development opportunities. The lowest mean stress score was reported for pressure to generate income or revenue (mean ± SD: 2.90 ± 1.40).

Perceived stress related to time-consuming administrative tasks differed significantly between age groups. Veterinarians aged 23–34 years reported the lowest stress levels (mean ± SD: 2.93 ± 1.12), whereas respondents aged ≥45 years reported the highest levels (mean ± SD: 3.91 ± 0.79) (Kruskal–Wallis: *H*(2) = 16.997, *p* < 0.0001) (Supplementary Table S2). Respondents aged 35–44 years reported the greatest perceived limitations in professional development opportunities (mean ± SD: 3.56 ± 1.19), whereas those aged ≥45 years reported the lowest levels (mean ± SD: 2.88 ± 1.01) (Kruskal–Wallis: *H*(2) = 7.202, *p* < 0.05).

Women reported higher perceived stress levels than men for several individual stressors (Supplementary Table S3). For work–life balance and working hours, female veterinarians reported higher stress (mean ± SD: 3.80 ± 1.11) than men (mean ± SD: 3.16 ± 1.11) (Mann–Whitney: *U* = 860.5, *p* < 0.05). Stress related to cooperation and communication with animal owners was also higher among women (mean ± SD: 3.76 ± 1.03) than among men (mean ± SD: 3.12 ± 1.17) (Mann–Whitney: *U* = 854.0, *p* < 0.01). Women also reported significantly greater strain when handling difficult medical cases (mean ± SD: 3.76 ± 1.10) than men (mean ± SD: 3.16 ± 0.90) (Mann–Whitney: *U* = 819.0, *p* < 0.01). A similar pattern was observed for time management and task scheduling, with women reporting higher stress (mean ± SD: 3.52 ± 1.05) than men (mean ± SD: 3.08 ± 0.81) (Mann–Whitney: *U* = 933.5, *p* < 0.05). Women also reported greater stress related to discussing payments with clients (mean ± SD: 3.65 ± 1.21) than men (mean ± SD: 2.92 ± 0.95) (Mann–Whitney: *U* = 791.5, *p* < 0.01).

Significant differences between employed veterinarians and practice owners were observed for time-consuming administrative tasks (Supplementary Table S4). Employers/practice owners reported higher stress (mean ± SD: 3.74 ± 1.03) than employed veterinarians (mean ± SD: 3.23 ± 1.16) (Mann–Whitney: *U* = 1.095.5, *p* < 0.05).

Perceived stress related to euthanasia decisions differed between veterinarians working ≤40 h and those working >40 h per week (Supplementary Table S5). Respondents working more than 40 h per week reported greater emotional or ethical stress related to euthanasia decisions (mean ± SD: 2.95 ± 1.27) than those working ≤40 h (mean ± SD: 2.49 ± 1.04) (Mann–Whitney: *U* = 1549.5, *p* < 0.05).

Significant differences across professional experience groups were observed for several stress-related factors (Supplementary Table S6). Perceived stress related to communication within the team differed according to years of professional experience. Veterinarians with 11–20 years of experience reported the highest perceived strain (mean ± SD: 4.11 ± 0.90), whereas respondents with 0–3 years of experience reported the lowest (mean ± SD: 3.09 ± 1.20) (Kruskal–Wallis: *H*(3) = 9.710, *p* < 0.05). A particularly pronounced difference between experience groups was observed for time-consuming administrative tasks, such as telephone calls and appointment coordination. Veterinarians with more than 20 years of experience reported the highest stress levels (mean ± SD: 4.00 ± 0.76) whereas early-career veterinarians with 0–3 years of experience reported the lowest (mean ± SD: 2.84 ± 1.08) (Kruskal–Wallis: *H*(3) = 21.335, *p* < 0.0001). Significant differences were also found for perceived limitations in professional development opportunities. Veterinarians with 0–3 years of experience reported the greatest perceived limitations (mean ± SD: 3.52 ± 1.02), whereas those with more than 20 years of experience reported the lowest levels (mean ± SD: 2.83 ± 0.93) (Kruskal–Wallis: *H*(3) = 8.341, *p* < 0.05).

Several stress-related items differed across the three self-reported mental health groups (Supplementary Table S7). For stress related to discussing payments with clients, respondents with satisfactory mental health reported the highest score (mean ± SD: 3.87 ± 1.24), compared with those reporting very good or good mental health (mean ± SD: 3.36 ± 1.11) and those reporting poor or very poor mental health (mean ± SD: 3.07 ± 1.27) (Kruskal–Wallis: *H*(2) = 7.726, *p* < 0.05). For pressure to generate income or revenue, respondents reporting poor or very poor mental health had the highest score (mean ± SD: 3.86 ± 1.41), whereas those reporting very good or good mental health had the lowest (mean ± SD: 2.48 ± 1.25) (Kruskal–Wallis: *H*(2) = 14.691, *p* < 0.001).

### 3.3. Associations Between Work-Related Stressors and Professional Decision-Making

**Figure 3:**
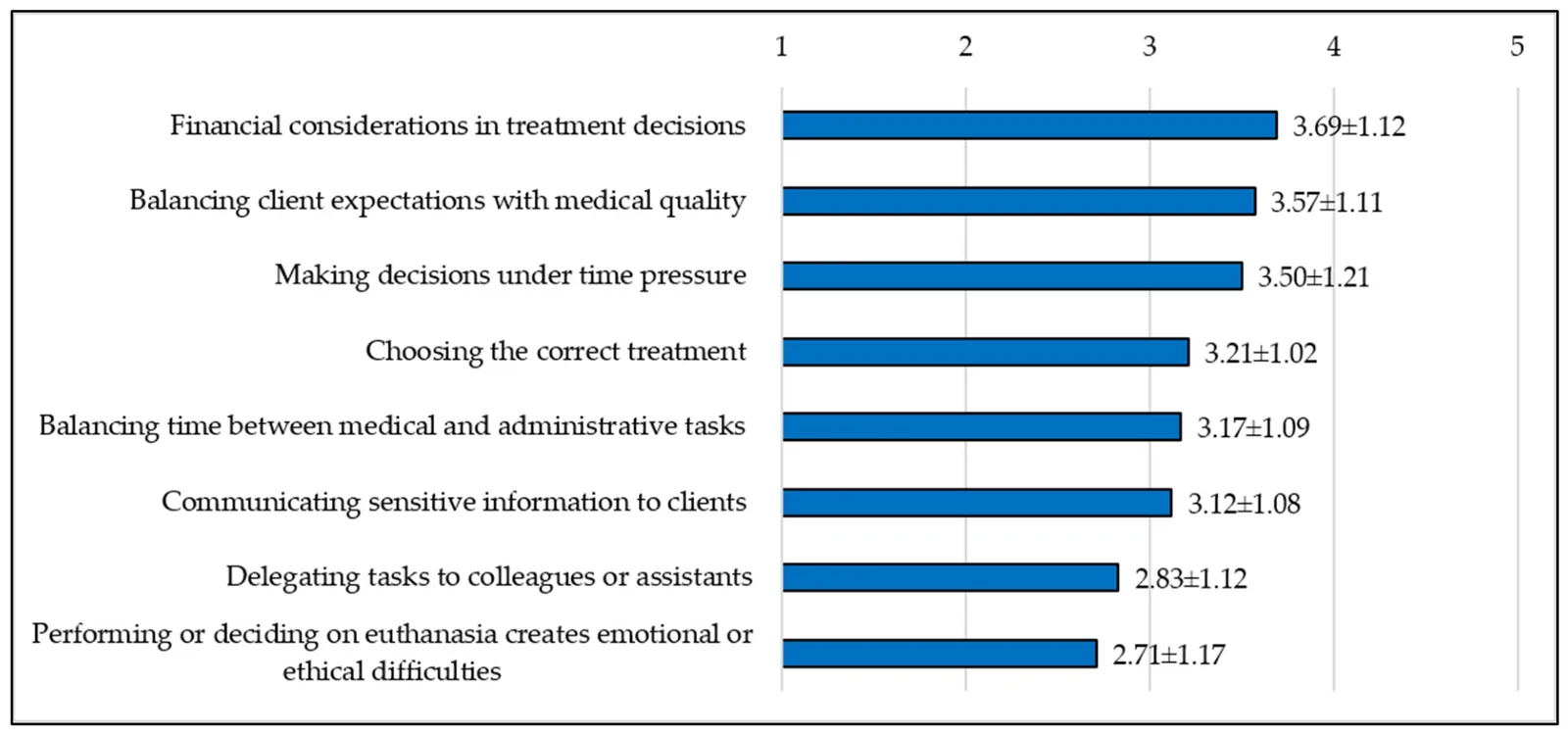
Veterinarians’ perceptions of factors influencing daily professional decision-making, ranked by mean score (mean ± SD; *n* = 126). Responses were given on a five-point Likert scale, where 1 = strongly disagree, 2 = disagree, 3 = neither agree nor disagree, 4 = agree, and 5 = strongly agree.

The analysis showed that various work-related stressors were perceived to influence veterinarians’ daily decision-making to differing degrees (Figure 3). Financial considerations in treatment decisions had the highest mean score (mean ± SD: 3.69 ± 1.12), followed by balancing client expectations with the provision of high-quality medical care and making decisions under time pressure. Lower mean scores were observed for delegating tasks (mean ± SD: 2.83 ± 1.12) and emotional or ethical difficulties related to euthanasia (mean ± SD: 2.71 ± 1.17).

Perceived stress related to selecting the appropriate treatment differed across age groups. Veterinarians aged 23–34 years reported the highest score (mean ± SD: 3.47 ± 1.11), followed by those aged 35–44 years (mean ± SD: 3.12 ± 0.88) and those aged ≥45 years (mean ± SD: 2.88 ± 0.88) (Kruskal–Wallis: *H*(2) = 8.090, *p* < 0.05). Balancing time between medical and administrative tasks also differed significantly across age groups (Kruskal–Wallis: *H*(2) = 10.288, *p* < 0.01). Veterinarians aged 23–34 years reported the lowest score (mean ± SD: 2.91 ± 1.08), whereas those aged ≥45 years reported the highest (mean ± SD: 3.68 ± 0.95).

Women reported greater perceived stress than men when balancing client expectations with medical standards (mean ± SD: 3.77 ± 1.01 vs. 2.76 ± 1.17) (Mann–Whitney: *U* = 670.5, *p* < 0.0001). A similar difference was observed for financial considerations in treatment decisions, with women reporting higher scores than men (mean ± SD: 3.89 ± 1.00 vs. 2.88 ± 1.24) (Mann–Whitney: *U* = 689.5, *p* < 0.0001).

Employed veterinarians reported greater stress related to selecting the appropriate treatment (mean ± SD: 3.34 ± 1.03) than practice owners (mean ± SD: 2.84 ± 0.90) (Mann–Whitney: *U* = 1084.5, *p* < 0.05). By contrast, employers perceived balancing time between medical and administrative tasks as more challenging (mean ± SD: 3.58 ± 1.12) than employed veterinarians (mean ± SD: 3.03 ± 1.06) (Mann-Whitney: *U* = 1080.5, *p* < 0.05).

Perceived stress related to selecting the appropriate treatment also differed across professional experience groups. Veterinarians with 0–3 years of experience reported the highest score (mean ± SD: 3.59 ± 1.04), whereas those with 11–20 years of experience reported the lowest (mean ± SD: 2.89 ± 0.90) (Kruskal–Wallis: *H*(3) = 10.631, *p* < 0.05). Balancing time between medical and administrative tasks also differed across experience groups. Veterinarians with more than 20 years of experience reported the highest perceived burden (mean ± SD: 3.79 ± 0.90), whereas those with 0–3 years of experience reported the lowest (mean ± SD: 2.84 ± 1.12) (Kruskal–Wallis: *H*(3) = 13.324, *p* < 0.01).

Several decision-making-related items differed across self-reported mental health groups. For performing or deciding on euthanasia, respondents reporting satisfactory mental health had the highest score (mean ± SD: 3.04 ± 1.21), whereas those reporting very good or good mental health had a lower score (mean ± SD: 2.46 ± 1.11) (Kruskal–Wallis: *H*(2) = 6.908, *p* < 0.05). For selecting the appropriate treatment, respondents reporting poor or very poor mental health had the highest score (mean ± SD: 3.50 ± 1.02), followed by those reporting satisfactory mental health (mean ± SD: 3.49 ± 0.97) and very good or good mental health (mean ± SD: 2.97 ± 1.00) (Kruskal–Wallis: *H*(2) = 8.596, *p* < 0.05).

A similar pattern was observed for making decisions under time pressure. Mean scores increased from respondents reporting very good or good mental health (mean ± SD: 3.24 ± 1.21) to those reporting satisfactory mental health (mean ± SD: 3.73 ± 1.12) and were highest among those reporting poor or very poor mental health (mean ± SD: 4.00 ± 1.30) (Kruskal–Wallis: *H*(2) = 7.817, *p* < 0.05). Stress related to balancing client expectations with the provision of high-quality medical care was highest among respondents reporting poor or very poor mental health (mean ± SD: 4.29 ± 1.07), compared with those reporting very good or good mental health (mean ± SD: 3.30 ± 1.07) (Kruskal–Wallis: *H*(2) = 13.000, *p* < 0.01). Financial considerations in treatment decisions also differed across mental health groups, with respondents reporting satisfactory mental health having the highest score (mean ± SD: 4.04 ± 1.00) (Kruskal–Wallis: *H*(2) = 8.921, *p* < 0.05).

### 3.4. Mental Health and Well-Being Outcomes

A substantial proportion of respondents indicated stress-related symptoms in the self-reported symptom checklist. Sleep disturbances were the most frequently reported item (63.5%), followed by burnout symptoms (31.7%) and anxiety (26.2%). Regarding psychological help-seeking, 61.9% of respondents reported that they had never sought professional support for work-related stress. However, 19.0% reported having sought such support, and an additional 19.0% had considered doing so but had not followed through.

Animal suffering and death also had a considerable perceived emotional impact. A total of 39.7% of respondents stated that animal suffering and death affected their mental well-being, whereas 42.9% reported no such effect. Work-related rumination outside working hours was highly prevalent: 56.3% of respondents reported that they often continued thinking about work-related decisions during their free time, while another 40.5% indicated that this depended on the situation.

Recovery from work-related stress during annual leave varied considerably. Nearly one-third (31.7%) required more than five days to experience relief, whereas 29.4% reported needing 1–2 days and 23.8% needed 3–5 days. A further 7.1% reported experiencing no relief from work-related stress during annual leave.

The availability of workplace psychological support was limited. Only 3.2% of respondents reported that such support was available and that they had used it, while 15.9% indicated that support was available but they had not used it. Among those reporting that no support was available, 27.0% expressed a desire for such services, whereas 35.7% stated that they did not feel the need for them. An additional 18.3% were unsure whether psychological support was available at their workplace. The full distribution of responses for these variables is shown in Table 2.

**Figure 4:**
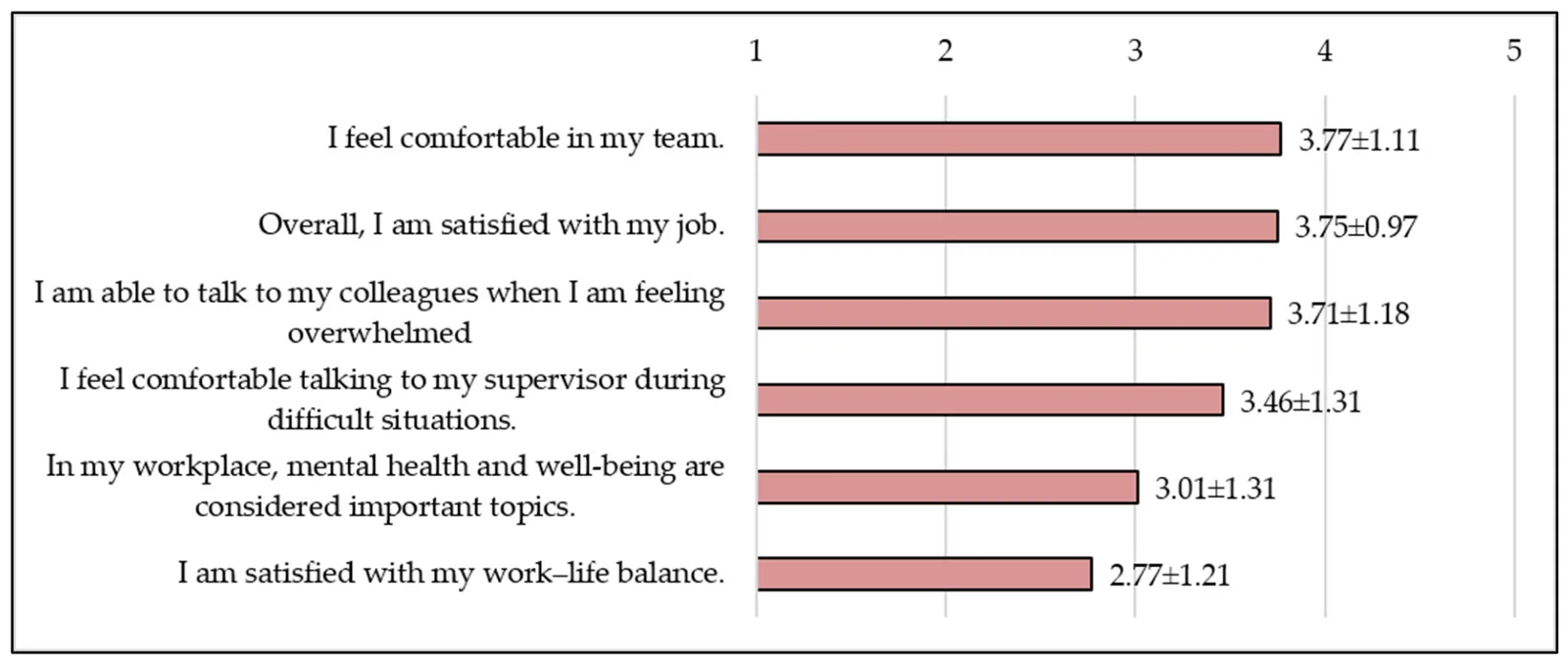
Veterinarians’ perceptions of social support, job satisfaction, and workplace well-being (mean ± SD; *n* = 126). Responses were given on a five-point Likert scale, where 1 = strongly disagree, 2 = disagree, 3 = neither agree nor disagree, 4 = agree, and 5 = strongly agree.

The mean scores for indicators related to social support, workplace well-being, and job satisfaction are shown in Figure 4. Respondents reported the highest agreement with the statement that they felt comfortable within their team (mean ± SD: 3.77 ± 1.11), followed by overall job satisfaction (mean ± SD: 3.75 ± 0.97). Perceived social support was also relatively strong, with respondents indicating that they could talk to colleagues when feeling overwhelmed and felt comfortable speaking to their supervisors in difficult situations. Lower scores were observed for perceived workplace recognition of the importance of mental health and well-being (mean ± SD: 3.01 ± 1.31) and satisfaction with work–life balance (mean ± SD: 2.77 ± 1.21).

Women reported lower overall job satisfaction than men (mean ± SD: 3.66 ± 0.97 vs. 4.12 ± 0.88) (Mann–Whitney: *U* = 936.5, *p* < 0.05). Practice owners reported greater agreement that mental health and well-being were regarded as important topics in their workplaces (mean ± SD: 3.48 ± 1.29) than employed veterinarians (mean ± SD: 2.85 ± 1.28) (Mann–Whitney: *U* = 1067.5, *p* < 0.05). Overall job satisfaction was also higher among employers (mean ± SD: 4.13 ± 0.96) than employed veterinarians (mean ± SD: 3.63 ± 0.95) (Mann–Whitney: *U* = 1026.5, *p* < 0.01).

Weekly working hours were also associated with satisfaction with work–life balance. Veterinarians working more than 40 h per week reported lower satisfaction (mean ± SD: 2.55 ± 1.21) than those working ≤ 40 h (mean ± SD: 3.02 ± 1.18) (Mann–Whitney: *U* = 1561.5, *p* < 0.05). Veterinarians with more than 28 days of annual leave reported greater comfort within their team (mean ± SD: 3.71 ± 1.19) than those with 28 days or fewer (mean ± SD: 3.22 ± 1.37) (Mann–Whitney: *U* = 1579.0, *p* < 0.05) (Supplementary Table S8).

The most pronounced differences across self-reported mental health groups were observed for indicators related to well-being and job satisfaction. Satisfaction with work–life balance was lowest among respondents reporting poor or very poor mental health (mean ± SD: 1.43 ± 0.65), followed by those reporting satisfactory mental health (mean ± SD: 2.38 ± 1.09, and was highest among those reporting very good or good mental health (mean ± SD: 3.31 ± 1.06) (Kruskal–Wallis: *H*(2) = 37.270, *p* < 0.0001).

Similarly, perceived comfort within the team was lowest among respondents reporting poor or very poor mental health (mean ± SD: 2.86 ± 1.17), followed by those reporting satisfactory mental health (mean ± SD: 3.71 ± 0.90), and highest among those reporting very good or good mental health (mean ± SD: 4.00 ± 1.14) (Kruskal–Wallis: *H*(2) = 13.394, *p* < 0.0001).

Substantial differences were also observed in perceived access to interpersonal support. Respondents reporting poor or very poor mental health reported the lowest agreement that they could talk to colleagues when feeling overwhelmed (mean ± SD: 2.79 ± 0.98), followed by those reporting satisfactory mental health (mean ± SD: 3.42 ± 1.16) and those reporting very good or good mental health (mean ± SD: 4.09 ± 1.08) (Kruskal–Wallis: *H*(2) = 20.031, *p* < 0.0001). A similar pattern was observed for perceived comfort when speaking to supervisors (Kruskal–Wallis: *H*(2) = 23.259, *p* < 0.0001).

Strong differences were also observed in the extent to which respondents perceived mental health and well-being as important workplace topics. Respondents reporting poor or very poor mental health had the lowest agreement (mean ± SD: 2.00 ± 1.04), followed by those reporting satisfactory mental health (mean ± SD: 2.71 ± 1.24) and those reporting very good or good mental health (mean ± SD: 3.42 ± 1.25) (Kruskal–Wallis: *H*(2) = 17.646, *p* < 0.0001).

Overall job satisfaction also differed substantially across mental health groups. Respondents reporting poor or very poor mental health had the lowest score (mean ± SD: 2.50 ± 1.02), whereas those reporting very good or good mental health had the highest (mean ± SD: 4.16 ± 0.81) (Kruskal–Wallis: *H*(2) = 35.003, *p* < 0.0001).

## 4. Discussion

### 4.1. Multidimensional Occupational Stress and Mental Health Burden Among Veterinarians Working in Germany

The present cross-sectional study indicates that veterinarians working in Germany experience occupational stress as a multidimensional burden, shaped by the combined effects of working time, work–life balance, emotionally demanding professional situations, client or communication, financial decision-making, and organizational workload. Although the findings are exploratory and based on a convenience sample, they are consistent with recent cross-national European evidence suggesting that several stressors are shared across veterinary populations, while others, such as the emotional burden associated with euthanasia or the availability of workplace support, may vary according to national and organizational context [16]. The findings are largely consistent with previous German and international studies highlighting the relevance of working hours, work–life balance, workload, administrative burden, client communication, and emotional demands in veterinary occupational stress [2,3,8,9,10,18,22,34,35,36,37]. The specific contribution of the present study is therefore not the identification of these stressors as entirely new factors, but their combined assessment together with professional decision-making, self-reported mental health indicators, job satisfaction, and workplace psychological support within one exploratory German sample.

In the present study, work–life balance and working hours emerged as the most prominent occupational stressors (mean ± SD: 3.67 ± 1.14). More than half of the respondents worked more than 40 h per week, and 82.5% reported some form of night, weekend, or on-call duty. These findings are consistent with international research that has repeatedly identified long working hours, irregular schedules, and insufficient recovery time as major contributors to burnout, emotional exhaustion, and reduced job satisfaction in veterinary profession [34,38]. They also align with scoping reviews and German studies showing that workload, time pressure, overcommitment, and maladaptive work-related behavioral patterns are central elements of occupational strain among veterinarians [2,8,9,10]. In a recent German survey of employed veterinarians, 22.1% of respondents reported working more than 10 h per day at least once per week, and approximately 40% reported being unable to take the legally required minimum 30-min break at least once per week [39]. These findings further support the interpretation that work–life balance should not be considered a secondary or merely lifestyle-related issue, but rather a core component of occupational health and professional sustainability within the veterinary profession [2,18,38,39]. Recent German evidence also supports the relevance of work-related behavioral patterns and overcommitment. Thielmann et al. [8] reported that 61.1% of veterinarians working in Germany showed a work-related risk pattern, and that the burnout-related Pattern B was the most common, affecting 40.3% of respondents. Similarly, Böckelmann et al. [10] found that younger veterinarians were more likely to show high overcommitment and higher levels of cognitive and emotional irritation, whereas older veterinarians reported comparatively lower levels of overcommitment and stress.

Administrative workload was another important source of stress, particularly among older and more experienced veterinarians. Respondents aged ≥45 years reported the highest perceived burden associated with time-consuming administrative tasks, and veterinarians with more than 20 years of professional experience also reported relatively high levels stress related to these tasks. This pattern is plausible, as increasing professional experience may be accompanied by greater responsibility, practice management tasks, economic decision-making, documentation duties, and staff coordination. Previous studies from Germany and Austria have similarly highlighted administrative burden, bureaucracy and practice-management responsibilities as relevant occupational stressors among veterinarians [2,8,9,10,18,35,40]. German data on employed veterinarians also indicate that working conditions, salary, corporate versus owner-managed employment structures, and compliance with working-time regulations remain relevant to job satisfaction and retention [39]. However, because the present study did not directly assess the specific managerial, ownership, or supervisory responsibilities of the respondents, this explanation for the greater administrative burden reported by older and more experienced participants remains tentative.

Interpersonal and emotional demands also played a central role in the stress profile of the respondents. Communication with animal owners, handling complex medical cases, and discussing treatment costs were perceived as important sources of strain. These findings are consistent with earlier studies identifying client communication, emotionally charged consultations, financial discussions, unrealistic expectations and difficult clinical situations as significant psychosocial stressors in veterinary work [2,3,9,41]. The importance of these factors is particularly relevant in small animal practice, where frequent and direct interaction with animal owners may intensify emotional labor and increase the burden associated with client expectations, complaints, and cost-related conflicts [2,3,9,41]. German research on official veterinarians also indicates that psychosocial strain is not restricted to private clinical practice, as official veterinarians may also experience high demands, exhaustion, and exposure to offensive behavior during their work [22].

Financial considerations in treatment decisions represented the highest-rated decision-making-related stressor (mean ± SD: 3.69 ± 1.12). This finding is consistent with research showing that economic constraints can create moral discomfort, increase pressure in decision-making, and generate concerns about criticism [41]. In clinical veterinary work, treatment decisions often require balancing medical possibilities, prognosis, animal welfare, client expectations, and the owner’s financial capacity. Such situations may lead to moral stress when veterinarians believe that a particular course of care would be preferable but are unable to provide or recommend it because of external limitations. This supports the interpretation that occupational stress in veterinary medicine is not only a workload issue, but also involves ethical, professional, and decision-making demands [2,3,9,41].

The mental health-related findings further underline the relevance of occupational stress in this population. In the self-reported symptom checklist, sleep disturbances were indicated by 63.5% of respondents, burnout symptoms by 31.7%, and anxiety by 26.2%. Because these symptoms were assessed using a study-specific checklist without a specified time frame, these percentages should not be interpreted as clinical diagnoses or prevalence estimates. Nevertheless, they are consistent with the broader international literature describing veterinarians as a professional group at increased risk of psychological distress, burnout, anxiety, depression, and suicidal ideation [34]. Scoping reviews and cross-sectional studies have repeatedly reported considerable levels of anxiety, depression and burnout among veterinarians [2,3,10,42]. For example, previous research reported that approximately 32% of Canadian veterinarians were classified as likely to have anxiety and 9% as likely to have depression, while German studies indicate that depressive symptoms may be substantially more frequent among veterinarians than in the general population [2]. In a large German study, high overcommitment and low reward were identified as important predictors of depressive symptoms, suicidal ideation, and suicide risk among veterinarians [43]. Research on moral distress and burnout also suggests that severe psychological distress, depression, and suicidal thoughts occur more frequently in veterinary populations than in many comparison groups [41].

A particularly important finding was the discrepancy between the frequency of stress-related symptoms and the use or availability of psychological support. Despite the high proportion of respondents reporting sleep disturbances, burnout symptoms, or anxiety, only 19.0% had sought psychological help for work-related stress. A further 19.0% had considered seeking help but had not done so. Workplace psychological support was rarely available or used, with only 3.2% reporting that such support existed and that they had used it. This pattern is consistent with international studies describing barriers to help-seeking, including stigma, limited access to services, time constraints, professional culture, and insufficient organizational support [15]. In a large U.S. survey, 59% of veterinarians with serious psychological distress reported that they were not currently receiving mental health treatment [4]. The present findings therefore suggest a support gap in German veterinary workplaces, where psychological strain is common but structured workplace-based support remains limited.

Overall, the findings indicate that working conditions, emotional demands, decision-making pressure, and organizational support are closely associated with the mental health and job satisfaction of veterinarians in Germany [34,35]. The study identified patterns consistent with those described in the international literature, but also adds Germany-specific exploratory evidence by examining daily stressors, professional decision-making, mental health symptoms, job satisfaction, and workplace support within the same survey. Together with recent German studies on work-related behavioral patterns, overcommitment, psychosocial working conditions, and employed veterinarians’ working conditions [8,10,22,39], the present findings suggest that improving veterinary well-being requires more than individual stress-management strategies. Structural and organizational measures addressing workload, administrative burden, predictable scheduling, communication challenges, ethical decision-making, and accessible mental health support may also be needed.

### 4.2. Subgroup Differences in Perceived Occupational Stress

The exploratory subgroup analyses suggest that perceived occupational stress differed according to gender, age, professional experience, employment status, working hours, and current mental health status. These differences indicate that occupational stress may not be experienced uniformly within the veterinary profession, but may vary according to career stage, professional role, workplace structure, and mental health-related vulnerability. However, these findings should be interpreted cautiously because the subgroup analyses were predominantly univariable and potential confounding by demographic and occupational factors cannot be excluded [8,10,22,39,44]. It should also be noted that several demographic and occupational variables were likely interrelated. For example, age and professional experience are closely connected, while professional experience may also be associated with employment status, practice ownership, administrative responsibilities, and working-time arrangements. Gender may also be unevenly distributed across fields of veterinary activity or employment contexts. Because the present analyses were primarily univariable, the potential influence of these interrelationships could not be fully accounted for, and subgroup-specific findings should therefore be interpreted as descriptive and hypothesis-generating rather than as independent effects [8,10,22,39].

Women reported higher perceived stress levels across several domains, including work–life balance, communication with animal owners, difficult medical cases, time management, and financial discussions. This pattern is consistent with international studies in which female veterinarians more frequently reported emotional exhaustion, anxiety, work–life conflict, and other indicators of psychological strain [3,9,41]. Similar patterns have also been described in the broader healthcare literature, where women have been found to report empathy-related stress and internalizing symptoms more frequently [2,44]. Several explanations have been proposed, including differences in emotional labor, work–life expectations, social role expectations, help-seeking behavior, and willingness to report psychological symptoms [2,41,45]. However, the present cross-sectional study cannot determine the mechanisms underlying these gender differences. Therefore, the findings should be interpreted as exploratory evidence of gender-related variation in perceived occupational stress, rather than as evidence of causal pathways.

Age and professional experience also appeared to be associated with the type of stress experienced. Veterinarians with limited professional experience, particularly those with 0–3 years of professional experience, reported greater stress related to clinical decision-making, including the selection of appropriate treatment options. In contrast, older and more experienced veterinarians reported greater stress associated with administrative burden. This suggests that the dominant sources of stress may shift over the professional life course. Early-career veterinarians working in clinical settings may experience greater uncertainty, lower autonomy, and higher pressure when making clinical decisions, whereas experienced veterinarians may increasingly face managerial, administrative, and economic responsibilities. This interpretation is consistent with previous findings showing that little professional experience, limited autonomy, and work overload may increase stress in the early stages of a veterinary career [34]. German studies also support this career-stage interpretation. Thielmann et al. [8] reported age-related differences in work-related behavioral and experimental patterns among veterinarians working in Germany, while Böckelmann et al. [10] found that younger veterinarians were more affected by overcommitment and cognitive and emotional irritation. At the same time, administrative and organizational responsibilities may become more prominent with increasing seniority, ownership responsibilities, and professional experience [2,3,8,9,10,18,19,35,40].

Employment status further contributed to differences in perceived stress. Practice owners appeared to be more affected by administrative workload, while employed veterinarians reported greater stress in relation to clinical decision-making. This distinction may reflect different role expectations within veterinary workplaces. Self-employed veterinarians and practice owners are responsible not only for clinical care, but also for organizational management, staff coordination, financial planning, client flow, and regulatory or administrative obligations [9,10,24]. In contrast, employed veterinarians working in clinical settings may be more exposed to direct patient care, clinical uncertainty, unclear responsibilities, or limited influence over resources and decision-making processes [10,40]. Similar patterns have been reported in German and Austrian studies, where self-employed veterinarians more often reported stress related to practice management, bureaucracy, and economic pressure, whereas employees were more affected by clinical errors, limited autonomy, and workplace-level constraints [9,24]. Recent German evidence on employed veterinarians further highlights that working conditions, salary, compliance with working-time regulations, and job satisfaction remain closely connected in this professional group [39].

Working hours represented another important subgroup-related factor. Veterinarians working more than 40 h per week reported lower satisfaction with their work–life balance, which is consistent with the broader veterinary literature showing that long working weeks, frequent night or weekend shifts, and on-call duties are associated with psychological stress, burnout, demoralization, and impaired recovery [2,7,9,15,24]. This is also consistent with the present finding that work–life balance and working hours were the highest-rated stressors overall. Recent German data on employed veterinarians also indicate that long working days and insufficient breaks remain relevant problems in veterinary employment, further supporting the importance of working-time organization for occupational health and retention [39]. These results indicate that long working hours may not only increase immediate workload, but may also reduce opportunities for recovery, family life, and psychological detachment from work, thereby contributing to lower well-being and job satisfaction [2,7,9,18,38,39].

Current mental health status was strongly associated with perceived occupational stress. Respondents with poorer perceived mental health reported higher stress in several areas, particularly time pressure, client expectations, financial conflicts, treatment decision-making, and a lack of workplace support. This pattern is consistent with previous studies showing that key stressors in veterinary medicine are associated with negative psychological outcomes [9]. However, because of the cross-sectional design, the direction of this relationship cannot be determined: poorer mental health may increase the perception of occupational stress, while occupational stress may also adversely affect mental health. Financial pressure may be especially relevant, as economic constraints in treatment decisions can intensify moral stress and have been linked to burnout and suicidal thoughts [9,19,41]. In Germany, high overcommitment and low reward have also been identified as potential predictors of depressive symptoms, suicidal ideation, and suicide risk among veterinarians [43]. Similarly, high workload, time pressure, and demanding conversations with animal owners are repeatedly described as potential contributors to mental overload, emotional exhaustion, and reduced professional fulfilment [3,24,34]. Moral stress, defined as the internal conflict that arises when professional decisions are incompatible with one’s values or perceived responsibilities, is also associated with poorer mental health and an increased risk of burnout in veterinary medicine [19,41].

Taken together, these exploratory subgroup findings suggest that preventive measures may need to consider differences in career stage, professional role, employment context, working conditions, and self-reported mental health status. Early-career veterinarians may benefit from structured mentoring, supervision, and support in clinical decision-making, whereas experienced veterinarians and practice owners may require greater administrative support, workload redistribution, and organizational resources. Employed veterinarians may benefit from clearer working-time arrangements, predictable schedules, fair salary structures, and greater influence over workplace processes [8,10,18,39,40]. However, because the subgroup analyses were univariable and potential confounding cannot be excluded, these group-specific implications should be interpreted as tentative and hypothesis-generating rather than definitive recommendations. The findings also suggest that gender-related differences and poorer self-reported mental health should be considered when designing supportive workplace measures [2,3,9,41]. However, supportive measures should be based on individual and occupational needs rather than on gender alone. These results reinforce the need for workplace-level prevention strategies that are sensitive to professional role, career stage, employment context, and mental health-related vulnerability [9,10,18,39,43,46].

### 4.3. Practical Implications for Veterinary Workplaces, Education, and Professional Support

The findings of this study have several practical implications for veterinary workplaces, professional organizations, and veterinary education. The results suggest that occupational stress in veterinary medicine should not be approached solely as an individual coping problem, but also as a workplace and organizational issue. Long working hours, irregular schedules, emotional demands, administrative workload, client communication, and limited psychological support were all relevant components of the stress profile observed in this sample. Previous research has also shown that these factors may affect not only personal well-being, but also job satisfaction, motivation, professional performance, and the quality and sustainability of veterinary services [2,19].

A first implication concerns working-time organization. In the present study, work–life balance and working hours represented the highest-rated stressors, and more than half of the respondents worked more than 40 h per week. These findings underline the importance of more predictable and sustainable working-time models. Flexible scheduling, reliable rest periods, equitable distribution of weekend, night and on-call duties, and realistic workload planning may help reduce chronic strain and improve recovery opportunities [1,2]. This is also supported by recent German evidence showing that compliance with working-time regulations, long working days, insufficient breaks, and job satisfaction remain relevant issues among employed veterinarians [39].

A second implication concerns staffing, task distribution, and administrative relief. Administrative workload was especially burdensome for older, more experienced veterinarians and practice owners, suggesting that greater professional responsibility may be accompanied by substantial organizational and bureaucratic demands. Veterinary workplaces may therefore benefit from clearer delegation of administrative tasks, better use of trained veterinary assistants or other support staff, improved appointment and workflow management, and digital or organizational solutions that reduce unnecessary documentation burden. A more balanced distribution of clinical, administrative, and communication-related tasks may reduce overload and support both individual well-being and team functioning [2,47,48]. Recent German findings on employed veterinarians also suggest that workplace structure, salary, overtime, compliance with working-time regulations, and job satisfaction are closely connected, further supporting the need for workplace-level organizational improvements [39].

Communication and conflict management also deserve greater attention. The present findings showed that communication with animal owners, complex medical cases, and financial discussions were important sources of stress. In clinical settings, these situations often require veterinarians to balance medical judgement, animal welfare, owner expectations, and financial limitations. Training in client communication, conflict resolution, shared decision-making, and cost-related conversations could therefore be strengthened both in veterinary education and in continuing professional development [1,24,38]. Such training may help veterinarians manage emotionally charged consultations, clarify expectations at an earlier stage, and reduce the psychological burden associated with difficult interactions [1,24,38]. For veterinarians working outside clinical practice, comparable training in stakeholder communication, negotiation, and the management of professional conflict may also be relevant.

Workplace culture is another important area for intervention. Open communication within veterinary teams may help reduce isolation and provide emotional relief after difficult cases, client conflicts, mistakes, or ethically challenging decisions. Regular team discussions, structured debriefings, mentoring systems, and peer-support models may strengthen psychological safety and team cohesion [2,47,48,49]. These approaches may be particularly useful for early-career veterinarians, who in the present study reported greater stress related to treatment decisions, and for respondents with poorer self-reported mental health, who reported lower perceived support in several workplace-related domains. German research among official veterinarians also indicates that psychosocial demands, emotional demands, exhaustion, and exposure to offensive behavior may occur outside private clinical practice as well, suggesting that workplace-level support should be considered across different veterinary sectors [22].

The limited availability and use of psychological support represent a particularly important practical concern. Although stress-related symptoms were common, only a minority of respondents had sought psychological help, and workplace psychological support was rarely available or used. This suggests that mental health support in veterinary workplaces may still depend too heavily on individual initiative. Employers, professional chambers, and veterinary associations should consider developing easily accessible, confidential support options, including mentoring programs, peer-support initiatives, anonymous counselling services, and referral pathways to professional mental health care [1,2]. Such services should complement, rather than replace, improvements in working conditions and organizational structures. At the same time, mental health should be addressed openly as a legitimate occupational health concern rather than as an individual weakness [2].

Veterinary education also has an important preventive role. Mental health, stress management, resilience, communication skills, ethical decision-making, and self-care should be integrated more systematically into the veterinary curriculum and postgraduate training [1,2,10]. This is particularly relevant because mental health concerns and emotional distress may already emerge during veterinary education. Recent research among veterinary students has reported burnout, emotional distress, and suicidal thoughts in this population [50]. In addition, studies on veterinary students’ career motivations and career intentions suggest that many students enter the profession with strong intrinsic and animal-related motivations [51]. These motivations may later come into conflict with the economic, administrative, and emotional realities of clinical practice [51]. Preparing students for these realities may support more realistic expectations, the development of effective coping and communication skills, and more sustainable career development [50].

Overall, the findings indicate that improving veterinary well-being requires action at several levels. Individual stress-management skills are important, but they are unlikely to be sufficient without structural and organizational changes. Sustainable prevention should include healthier working-time models, adequate staffing, administrative support, communication training, accessible psychological support, and a workplace culture in which mental health can be discussed without stigma. Recent German studies on employed and official veterinarians further indicate that working conditions, organizational structure, psychosocial demands, and workplace support remain key issues across different veterinary employment contexts [22,39]. Such measures may contribute to healthier, more stable, and more satisfying veterinary careers, although their feasibility and effectiveness should be evaluated in future intervention studies [2,18,39,40].

### 4.4. Limitations

Several methodological limitations should be considered when interpreting the findings. First, participants were recruited through non-probability convenience sampling via online channels, veterinary social media platforms, and personal contacts. Therefore, the sample cannot be considered nationally representative of all veterinarians working in Germany. Response rates could not be calculated, and participation may have been influenced by self-selection bias. Veterinarians with stronger experiences, concerns, or opinions related to occupational stress, mental health, client communication, or work–life balance may have been more likely to participate. Conversely, veterinarians experiencing very high workload, severe psychological strain, or limited time may have been less likely to complete the questionnaire. The relatively high proportion of female respondents and small animal practitioners, as well as the uneven distribution across fields of veterinary work and federal states, may further limit the generalizability of subgroup-specific findings. In addition, several subgroup comparisons were based on small group sizes, including male respondents (*n* = 25) and respondents reporting poor or very poor mental health (*n* = 14). This may have reduced statistical power and increased the uncertainty of subgroup-specific estimates. Therefore, subgroup findings should be interpreted cautiously as exploratory and descriptive patterns rather than as robust evidence of group differences. Overall, the findings should be interpreted as exploratory and descriptive rather than as representative estimates for all veterinarians working in Germany.

Second, the data were based on self-reported questionnaire responses and may therefore be affected by recall bias, social desirability bias, current mood, and individual differences in response style. Mental health symptoms, stress levels, work–life balance, and job satisfaction were not assessed using clinical diagnostic interviews or objective workplace indicators. No objective data were available on actual working hours, sick leave, staff turnover, workload, patient outcomes or work performance. Consequently, the findings reflect perceived occupational stressors and self-reported mental health rather than clinically verified outcomes or objectively measured workplace conditions.

Third, although the questionnaire was developed based on previous literature and pilot-tested among veterinarians, it was not fully psychometrically validated. Established instruments such as the Copenhagen Burnout Inventory and the Job Content Questionnaire were reviewed during questionnaire development, but the final survey consisted of original items adapted to the veterinary context and the exploratory aims of the study. This approach allowed the questionnaire to capture specific stressors relevant to everyday veterinary work, including client communication, financial decision-making, administrative burden, and workplace support, but limited direct comparison with studies using standardized and validated composite measures. Nevertheless, the relevant item groups showed acceptable to very good internal consistency.

Fourth, the analyses were primarily exploratory and involved multiple subgroup comparisons across demographic and occupational variables. No formal correction for multiple comparisons was applied. Therefore, statistically significant findings should be interpreted cautiously as exploratory associations that may help generate hypotheses for future research rather than as confirmatory evidence. In addition, the analyses were mainly univariable, and residual confounding by factors such as professional field, workload intensity, income, staffing level, workplace culture, leadership style, previous mental health history, or availability of organizational support cannot be excluded.

Finally, the cross-sectional design prevents conclusions about temporal order or causality. The findings describe associations at one point in time but cannot determine whether specific working conditions were associated with subsequent deterioration in poorer mental health, whether respondents with poorer mental health perceived work-related stressors more strongly, or whether both processes occurred simultaneously. Longitudinal studies using larger, more representative samples, validated instruments, and multivariable analyses are needed to clarify these relationships and confirm the exploratory patterns identified here.

## 5. Conclusions

Compared with previous surveys, the specific contribution of the present study lies in the combined exploratory assessment of everyday occupational stressors, working conditions, professional decision-making factors, self-reported mental health indicators, job satisfaction, and workplace psychological support within the same sample of veterinarians working in Germany.

This study provides exploratory evidence that veterinarians working in Germany experience substantial occupational stress, particularly in relation to work–life balance, working hours, emotional and interpersonal demands, financial decision-making, and administrative workload. Self-reported stress-related symptoms, including sleep disturbances, burnout symptoms, and anxiety, were frequently indicated in the symptom checklist, while the availability and use of workplace psychological support appeared limited. These findings suggest that mental health and well-being in veterinary medicine cannot be addressed solely through individual coping strategies, but also require attention to structural and organizational measures.

The study also indicates that perceived stressors may differ according to gender, career stage, employment status, working hours, and current mental health status. These subgroup-related findings should be interpreted as tentative and hypothesis-generating, but they may suggest that early-career veterinarians, particularly those working in clinical settings, may benefit from stronger mentoring and support in clinical decision-making, whereas experienced veterinarians and practice owners may benefit from administrative relief and organizational support. The findings underline the importance of sustainable working-time models, adequate staffing, communication and conflict-management training, accessible psychological support, and a workplace culture in which mental health can be discussed without stigma. However, supportive measures should be tailored to individual needs, professional roles, and employment settings rather than applied uniformly across the profession.

Overall, the findings indicate that occupational stress among veterinarians working in Germany reflects a combination of professional, organizational, and individual factors. Improving veterinary well-being therefore requires coordinated measures that promote sustainable working conditions, organizational support, and accessible mental health resources. Future representative, longitudinal, and intervention-based studies are needed to confirm these findings and to evaluate measures aimed at improving working conditions, professional well-being, and long-term retention in the veterinary profession.

## Figures and Tables

**Table 1:** Sociodemographic and occupational characteristics of the respondents (*n* = 126).

Variable	Category	Number of Responses (*n* = 126)	Percentage of Responses (%)
Age	23–34 years	58	46.0%
	35–44 years	34	27.0%
	≥45 years	34	27.0%
Gender	Female	101	80.2%
	Male	25	19.8%
Field of veterinary activity	Small animals (dogs and cats)	90	71.4%
	Horses	30	23.8%
	Exotic animals (birds, reptiles, small mammals, etc.)	23	18.3%
	Cattle	16	12.7%
	Public veterinary service	4	3.2%
	Teaching, research, doctoral studies	4	3.2%
	Poultry	2	1.6%
	Pigs	1	0.8%
	Laboratory work	1	0.8%
	Other	3	2.4%
Employment status	Employee	95	75.4%
	Employer/practice owner	31	24.6%
Weekly working hours	≤40 h	59	46.8%
	>40 h	67	53.2%
Annual leave	≤28 days	64	50.8%
	>28 days	62	49.2%
Professional experience	0–3 years	44	34.9%
	4–10 years	35	27.8%
	11–20 years	18	14.3%
	>20 years	29	23.0%
On-call shifts	No	22	17.5%
	Yes, approx. once a month	31	24.6%
	Yes, 2–3 times per month	28	22.2%
	Yes, once a week	14	11.1%
	Yes, more than once a week	21	16.7%
Availability of an assistant during work	Always	19	15.1%
	Regularly	36	28.6%
	Sometimes	31	24.6%
	Rarely	24	19.0%
	Never	16	12.7%
Ability to manage time effectively during the working day	Very Good and Good	79	62.7%
	Satisfactory	40	31.7%
	Poor and Very Poor	7	5.6%
Self-reported current mental health	Very Good and Good	67	53.2%
	Satisfactory	45	35.7%
	Poor and Very Poor	14	11.1%

Note: Multiple responses were allowed for the field of veterinary activity; therefore, percentages may add up to more than 100%.

**Table 2:** Self-reported mental health-related experiences, emotional strain, recovery, and workplace support among veterinarians in Germany (*n* = 126).

Variable	Category	Number of Respondents (*n*)	Percentage of Respondents (%)
Stress-related symptoms	Sleep disturbances	80	63.5%
	Burnout symptoms	40	31.7%
	Anxiety	33	26.2%
	Depression	29	23.0%
	Panic attacks	23	18.3%
	Other	10	7.9%
Psychological help-seeking for work-related stress	No	78	61.9%
	Yes	24	19.0%
	Considered seeking help but did not follow through	24	19.0%
Perceived impact of animal suffering and death on mental well-being	No	54	42.9%
	Yes	50	39.7%
	Unsure	22	17.5%
Work-related rumination outside working hours	Yes, often	71	56.3%
	Depending on the situation	51	40.5%
	No, never	4	3.2%
Time required to recover from work-related stress during annual leave	Immediate relief	10	7.9%
	1–2 days	37	29.4%
	3–5 days	30	23.8%
	More than 5 days	40	31.7%
	No relief during annual leave	9	7.1%
Workplace psychological support	Yes, and I have used it	4	3.2%
	Yes, but I have not used it	20	15.9%
	No, and I do not feel the need for it	45	35.7%
	No, but I would like such support	34	27.0%
	Unsure whether support is available	23	18.3%

Note: Multiple responses were allowed for stress-related symptoms; therefore, the corresponding percentages may add up to more than 100%. Stress-related symptoms were assessed using a self-reported symptom checklist without a specified time frame, they should therefore be interpreted as self-reported indicators rather than clinical diagnoses or prevalence estimates. Other reported symptoms included headaches, migraines, high blood pressure, hair loss, gastritis, back pain, nightmares, and persistent fatigue.

## Data Availability

The original contributions presented in this study are included in the article/Supplementary Material. Further inquiries can be directed to the corresponding author.

## Supplementary Materials

The following supporting information can be downloaded at https://www.mdpi.com/article/10.3390/healthcare14172749/s1, Supplementary Questionnaire; Table S1: Internal consistency of predefined multi-item groups used in the questionnaire (*n* = 126); Table S2: Differences in perceived occupational stressors and mental health outcomes by age groups among veterinarians in Germany (*n* = 126); Table S3: Differences in perceived occupational stressors and mental health outcomes by gender among veterinarians in Germany (*n* = 126); Table S4: Differences in perceived occupational stressors and mental health outcomes by employment status among veterinarians in Germany (*n* = 126); Table S5: Differences in perceived occupational stressors and mental health outcomes by weekly working hours among veterinarians in Germany (*n* = 126); Table S6: Differences in perceived occupational stressors and mental health outcomes by professional experience among veterinarians in Germany (*n* = 126); Table S7: Differences in perceived occupational stressors and mental health outcomes by current mental health status among veterinarians in Germany (*n* = 126); Table S8: Differences between perceived occupational stressors and mental health outcomes by annual leave entitlement among veterinarians in Germany (*n* = 126).

## References

[1] Bartram D.J., Baldwin D.S. (2010). Veterinary surgeons and suicide: A structured review of possible influences on increased risk. Vet. Rec..

[2] Pohl R., Botscharow J., Böckelmann I., Thielmann B. (2022). Stress and strain among veterinarians: A scoping review. Ir. Vet. J..

[3] Stetina B.U., Krouzecky C. (2022). Reviewing a decade of change for veterinarians: Past, present and gaps in researching stress, coping and mental health risks. Animals.

[4] Nett R.J., Witte T.K., Holzbauer S.M., Elchos B.L., Campagnolo E.R., Musgrave K.J., Carter K.K., Kurkjian K.M., Vanicek C.F., O’Leary D.R. (2015). Risk factors for suicide, attitudes toward mental illness, and practice-related stressors among US veterinarians. J. Am. Vet. Med. Assoc..

[5] Volk J.O., Schimmack U., Strand E.B., Reinhard A., Hahn J., Andrews J., Probyn-Smith K., Jones R. (2024). Merck Animal Health Veterinary Team study reveals factors associated with well-being, burnout, and mental health among nonveterinarian practice team members. J. Am. Vet. Med. Assoc..

[6] Gardner D., Hini D. (2006). Work-related stress in the veterinary profession in New Zealand. N. Z. Vet. J..

[7] Connolly C., Norris K., Martin A., Dawkins S., Meehan C. (2022). A taxonomy of occupational and organisational stressors and protectors of mental health reported by veterinary professionals in Australasia. Aust. Vet. J..

[8] Thielmann B., Döring E., Pohl R., Böckelmann I. (2025). A cross-sectional study of work-related behaviour and experience patterns among German veterinarians in different age groups. Healthcare.

[9] Neubauer V., Gächter A., Probst T., Brühl D., Dale R., Pieh C., Humer E. (2024). Stress factors in veterinary medicine—A cross-sectional study among veterinary students and practicing vets in Austria. Front. Vet. Sci..

[10] Böckelmann I., Döring E., Pohl R., Thielmann B. (2025). Cognitive and emotional irritation in German veterinarians with different levels of overcommitment. Vet. Sci..

[11] Podpečan O., Hlebec V., Kuhar M., Kubale V., Jakovac Strajn B. (2025). Predictors of burnout and well-being among veterinarians in Slovenia. Vet. Sci..

[12] Džaja P., Severin K., Gašpar A., Zemljak I., Križek I., Butković I., Piplica A., Palić M. (2025). A survey of the impact of potential work-related stressors on mental health among veterinarians in Croatia. Vet. Stanica.

[13] Guntín S., López-Roel S., Isorna M., Fariña F. (2026). Mental health in Spanish veterinarians: Emotional exhaustion, affective symptomatology, and suicidal ideation. Eur. J. Investig. Health Psychol. Educ..

[14] Jansen W., Lockett L., Colville T., Uldahl M., De Briyne N. (2024). Veterinarian—Chasing a Dream Job? A Comparative Survey on Wellbeing and Stress Levels among European Veterinarians between 2018 and 2023. Vet. Sci..

[15] Máté M., Várnai C.H., Ózsvári L. (2025). A cross-national study on mental health, psychological distress and suicidal ideation among veterinarians in multiple European countries. Front. Vet. Sci..

[16] Máté M., Várnai C.H., Ózsvári L. (2026). Work-related stressors and their perceived impact on veterinary work and personal life: A multi-country European study. Vet. Sci..

[17] Diez E., Saur R., Máté M., Ózsvári L. (2026). Perceptions of feminization in the German veterinary profession. Acta Vet. Eurasia.

[18] Máté M., Schwarz E.S., Ózsvári L. (2025). Differences in income, workload, and job satisfaction among Hungarian and German veterinarians. BMC Vet. Res..

[19] Steffey M.A., Griffon D.J., Risselada M., Buote N.J., Scharf V.F., Zamprogno H., Winter A.L. (2023). A narrative review of the physiology and health effects of burnout associated with veterinarian-pertinent occupational stressors. Front. Vet. Sci..

[20] Gresele B.S., Pereira J.L., da Silva A.R.S., Scavacini K., Lyrio-Carvalho H.C., Ulisses S.M.V., da Silva Rosa A. (2026). Psychological distress as a central mediator of suicidal ideation among Brazilian veterinarians: A study of occupational stress, compassion fatigue, coping strategies, and workplace environment using structural equation modeling. Vet. World.

[21] Platt B., Hawton K., Simkin S., Dean R., Mellanby R.J. (2012). Suicidality in the veterinary profession: Interview study of veterinarians with a history of suicidal ideation or behavior. Crisis.

[22] Jensen K.C., Merle R. (2024). Survey on psychosocial conditions of official veterinarians in Germany: Comparison with other professions and differences between age groups, gender, and workplace characteristics. Animals.

[23] Dawson B.F.Y., Thompson N.J. (2017). The effect of personality on occupational stress in veterinary surgeons. J. Vet. Med. Educ..

[24] Harling M., Strehmel P., Schablon A., Nienhaus A. (2009). Psychosocial stress, demoralization and the consumption of tobacco, alcohol and medical drugs by veterinarians. J. Occup. Med. Toxicol..

[25] Obbarius N., Fischer F., Liegl G., Obbarius A., Rose M. (2021). A modified version of the transactional stress concept according to Lazarus and Folkman was confirmed in a psychosomatic inpatient sample. Front. Psychol..

[26] Akbari J., Akbari R., Shakerian M., Mahaki B. (2017). Job demand-control and job stress at work: A cross-sectional study among prison staff. J. Educ. Health Promot..

[27] Aronsson G., Theorell T., Grape T., Hammarström A., Hogstedt C., Marteinsdottir I., Skoog I., Träskman-Bendz L., Hall C. (2017). A systematic review including meta-analysis of work environment and burnout symptoms. BMC Public Health.

[28] De Hert S. (2020). Burnout in healthcare workers: Prevalence, impact and preventative strategies. Local Reg. Anesth..

[29] Meredith L.S., Bouskill K., Chang J., Larkin J., Motala A., Hempel S. (2022). Predictors of burnout among US healthcare providers: A systematic review. BMJ Open.

[30] IBM Corp (2017). IBM SPSS Statistics for Windows.

[31] Cohen J. (2009). Statistical Power Analysis for the Behavioral Sciences.

[32] George D., Mallery P. (2016). IBM SPSS Statistics 23 Step by Step: A Simple Guide and Reference.

[33] Bundestierärztekammer e.V. Tierärztestatistik 2025: Mehr Tierärzt:innen—Aber weniger Selbstständige. Presseinformation Nr. 7/2026, 1 June 2026. https://www.bundestieraerztekammer.de/d.php?id=9764.

[34] Silva C.R.D., Gomes A.A.D., Santos-Doni T.R.D., Antonelli A.C., Vieira R.F.D.C., Silva A.R.S.D. (2023). Suicide in veterinary medicine: A literature review. Vet. World.

[35] Hernández-Esteve I., Zumbado M., Henríquez-Hernández L.A. (2025). Burnout and mental health among veterinarians: The role of self-compassion and associated risk factors. Vet. Rec..

[36] Romão M.E., Beheshti S.R., Scoccianti S., Barello S. (2026). Veterinarian–Client Communication as a Driver of Burnout: A Scoping Review of Relational Risk and Protective Resources. Vet. Sci..

[37] Frippiat T., Deleu S., Timmenga F., Mastenbroek N. (2026). Exploratory study of wellbeing in Dutch veterinarians and the impact of work-related factors and inappropriate behaviour by clients and coworkers. Vet. Rec..

[38] Dürnberger C., Springer S. (2025). “I’m not just a vet, I’m also a human.” A qualitative interview study on boundary management between work and private life among small animal veterinarians. PLoS ONE.

[39] Jensen K.C., Wunderlich C., Steingräber L., Brandebusemeyer E. (2026). Survey on the working conditions, salary, and job satisfaction of employed veterinarians in Germany. Vet. Sci..

[40] Kersebohm J.C., Lorenz T., Becher A., Doherr M.G. (2017). Factors related to work and life satisfaction of veterinary practitioners in Germany. Vet. Rec. Open.

[41] Prato-Previde E., De Mori B., Colombo N., Pelosi A. (2024). Willing but unable: Moral distress and burnout in Italian veterinarians working with companion and farm animals. Animals.

[42] Mudry A., Truchot D., Andela M. (2025). Development and longitudinal validation of the Veterinary Stressors Questionnaire. Sci. Rep..

[43] Schwerdtfeger K.A., Glaesmer H., Bahramsoltani M. (2024). High overcommitment and low reward as potential predictors for increased depressive symptoms, suicidal ideation, and suicide risk in German veterinarians. PLoS ONE.

[44] Andela M. (2020). Burnout, somatic complaints, and suicidal ideations among veterinarians: Development and validation of the Veterinarians Stressors Inventory. J. Vet. Behav..

[45] Brscic M., Contiero B., Schianchi A., Marogna C. (2021). Challenging suicide, burnout, and depression among veterinary practitioners and students: Text mining and topics modelling analysis of the scientific literature. BMC Vet. Res..

[46] Christiansen S.V., Clausen T. (2026). Emotional demands and depression: The buffering role of positive work-related factors in clinical veterinary practice. Vet. Rec..

[47] Olson K., Sinsky C., Rinne S.T., Long T., Vender R., Mukherjee S., Bennick M., Linzer M. (2019). Cross-sectional survey of workplace stressors associated with physician burnout measured by the Mini-Z and the Maslach Burnout Inventory. Stress Health.

[48] Tawfik D.S., Sinha A., Bayati M., Adair K.C., Shanafelt T.D., Sexton J.B., Profit J. (2021). Frustration with technology and its relation to emotional exhaustion among health care workers: Cross-sectional observational study. J. Med. Internet Res..

[49] Wu Y., Wu M., Wang C., Lin J., Liu J., Liu S. (2024). Evaluating the prevalence of burnout among health care professionals related to electronic health record use: Systematic review and meta-analysis. JMIR Med. Inform..

[50] Várnai C.H., Ózsvári L., Máté M. (2026). A cross-sectional study on burnout, emotional distress, and suicidal thoughts among international and Hungarian veterinary students. J. Vet. Med. Educ..

[51] Szücs L., Fehérvári P., Ózsvári L. (2025). Predicting veterinary career intentions using motivational characteristics: A survey study among Hungarian students. Vet. Sci..

