# Supplementary material for: Occupational Stressors, Working Conditions and Self-Reported Mental Health Among Veterinarians in Germany: An Exploratory Cross-Sectional Survey
Source: Healthcare (Basel). 2026 Aug 28;14(17):2749. doi: 10.3390/healthcare14172749 (PMC13564705; doi:10.3390/healthcare14172749)
Supplement: Supplementary file 1 [file healthcare-14-02749-s001.zip › healthcare-4499340-supplementary.pdf]

Supplementary Materials

Supplementary Questionnaire

We are conducting my research on the impact of veterinary work-related stressors on veterinary activity and mental health among German veterinarians. Veterinary professionals are known to experience higher levels of work-related stress, which can significantly affect their mental health and job satisfaction. In my thesis, I aim to investigate the psychosocial working conditions, self-reported stress factors, and job satisfaction among veterinarians.

The data for this study will be collected using an anonymous questionnaire, and responses will be analyzed in aggregate form. Completing the questionnaire should take approximately 15 minutes.

Thank you very much for contributing to our understanding of veterinary mental health and workplace conditions!

Q 1: Which age group do you belong to?

- 23-34
- 35-44
- 45-54
- 55-64
- 65-74
- Older than 74

Q 2: What is your gender?

- Female
- Male

Q 3: In which federal state (*Bundesland*) is your current workplace located?

- Baden-Württemberg
- Bavaria (Bayern)
- Berlin
- Brandenburg
- Bremen
- Hamburg
- Hesse (Hessen)
- Mecklenburg-Western Pomerania (Mecklenburg-Vorpommern)
- Lower Saxony (Niedersachsen)
- North Rhine-Westphalia (Nordrhein-Westfalen)
- Rhineland-Palatinate (Rheinland-Pfalz)

- Saarland
- Saxony (Sachsen)
- Saxony-Anhalt (Sachsen-Anhalt)
- Schleswig-Holstein
- Thuringia (Thüringen)

Q 4: What field are you currently working in within veterinary medicine? (You may choose more than one answer!)

- Small animals (dogs and cats)
- Exotic animals (birds, reptiles, small mammals, etc.)
- Equine
- Cattle
- Pig
- Poultry
- Laboratory work
- Authorities/State Vet
- Teaching/Research/PhD
- Other: ___________________

Q 5: Are you employed or employer?

- Employed
- Employer

Q 6: On average, how many hours do you work per week in total?

- Less than 20 hours
- 20-30 hours
- 31-40 hours
- 41-50 hours
- 51-60 hours
- Over 60 hours

Q 7: How many vacation days are you granted per year?

- Less than 14 days
- 14-28 days
- 29-35 days
- 36-42 days
- More than 42 days

Q 8: Are you obligated to have shifts (e.g., emergency, night or weekend shifts and on-call duty?

- No

- Yes, cc. once a month
- Yes, 2-3x per month
- Yes, once a week
- Yes, more than once a week

Q 9: How many years of professional experience do you have?

- Less than 1 year
- 1-3 years
- 4-6 years
- 7-10 years
- 11-20 years
- 21-30 years
- More than 30 years

Q 10: Do you usually work with an assistant who significantly reduces your workload?

- Always
- Regularly
- Sometimes
- Rarely
- Never

Q 11: How would you rate your ability to manage your time effectively during the workday?

- Very good
- Good
- Satisfactory
- Poor
- Very poor

Q 12: How would you rate your current mental health?

- Very good
- Good
- Fair
- Poor
- Very poor

Q 13: Have you ever experienced any of the following due to work-related stress? (You may choose more than one answer!)

- Sleep disturbances
- Anxiety

- Depression
- Burnout Symptoms
- Panic attacks
- None of the above
- Other: ____________________

Q 14: Have you ever sought professional psychological help due to work-related stress?

- Yes
- No
- I considered it, but I did not follow through

Q 15: Does animal suffering or death in your daily work affect your mental well-being?

- Yes
- No
- I am not sure

Q 16: Do you often think about work-related decisions after working hours?

- Yes, often
- No, never
- It depends

Q 17: How much time do you usually need during your vacation to feel relief from work-related stress?

- I feel relief immediately
- 1-2 days
- 3-5 days
- More than 5 days
- I do not feel relief from work-related stress during my vacation.

Q 18: Do you feel that psychological support or supervision is available at your workplace?

- Yes, and I have used it
- Yes, but I have not used it
- No, but I would like such support
- No, and I do not feel the need for it
- I do not know / not sure

Please indicate how much do you agree that the following factors contribute to stress in your daily veterinary work.

Likert 1-5 scale: where 1 = strongly disagree, 2 = disagree, 3 = neither agree nor disagree, 4 = agree, 5 = strongly agree.

- Q 19: Work–life balance and working hours
- Q 20: Cooperation and communication with pet owners
- Q 21: Cooperation and communication with colleagues
- Q 22: Communication within the team
- Q 23: Handling difficult medical cases
- Q 24: Time management and scheduling tasks
- Q 25: Discussing payments with clients
- Q 26: Pressure to generate income/revenue
- Q 27: Time-consuming administrative tasks (e.g., phone calls, appointment coordination)
- Q 28: My own professional development

Please indicate how much do you agree that the following stress factors influence your daily decision-making at work.

Likert 1-5 scale: where 1 = strongly disagree, 2 = disagree, 3 = neither agree nor disagree, 4 = agree, 5 = strongly agree.

- Q 29: Performing or deciding on euthanasia creates emotional or ethical difficulties
- Q 30: Choosing the correct treatment
- Q 31: Balancing time between medical and administrative tasks
- Q 32: Communicating sensitive information to clients
- Q 33: Making decisions under time pressure
- Q 34: Balancing client expectations with medical quality
- Q 35: Financial considerations in treatment decisions
- Q 36: Delegating tasks to colleagues or assistants

Please indicate how much do you agree with the following statements.

Likert 1-5 scale: where 1 = strongly disagree, 2 = disagree, 3 = neither agree nor disagree, 4 = agree, 5 = strongly agree.

Q 37: I am satisfied with my work–life balance.

Q 38: I feel comfortable in my team.

Q 39: I am able to talk to my colleagues when I am feeling overwhelmed

Q 40: I feel comfortable talking to my supervisor during difficult situations.

Q 41: In my workplace, mental health and well-being are considered important topics.

Q 42: Overall, I am satisfied with my job.

Q 43: If you have any additional comments regarding anything mentioned in the questionnaire, please add them below:

____

Thank you for your valuable contribution!

Supplementary Table S1. Internal consistency of predefined multi-item groups used in the questionnaire (n = 126).

Multi-item group	Items included	Cronbach's α
**Daily work-related stressors (n=10)**	Work–life balance and working hours	0.743
	Cooperation and communication with pet owners	
	Cooperation and communication with colleagues	
	Communication within the team	
	Handling difficult medical cases	
	Time management and scheduling tasks	
	Discussing payments with clients	
	Pressure to generate income/revenue	
	Time-consuming administrative tasks (e.g., phone calls, appointment coordination)	
	Professional development opportunities	
**Professional decision-making factors (n=8)**	Performing or deciding on euthanasia creates emotional or ethical difficulties	0.794
	Choosing the correct treatment	
	Balancing time between medical and administrative tasks	
	Communicating sensitive information to clients	
	Making decisions under time pressure	
	Balancing client expectations with medical quality	
	Financial considerations in treatment decisions	
	Delegating tasks to colleagues or assistants	
**Job satisfaction and workplace well-being (n=6)**	I am satisfied with my work–life balance.	0.862
	I feel comfortable in my team.	
	I am able to talk to my colleagues when I am feeling overwhelmed.	
	I feel comfortable talking to my supervisor during difficult situations.	
	In my workplace, mental health and well-being are considered important topics.	
	Overall, I am satisfied with my job.	

Supplementary Table S2. Differences in perceived occupational stressors and mental health outcomes by age groups among veterinarians in Germany (n = 126).

Stressors and Statements (n=24)	Age group			Pearson's chi-square (χ2) test			Cramer's V value	Kruskal–Wallis test				
	23-34 years (n=58)	35-44 years (n=34)	Over 44 years (n=34)	X-squared	df	p value		Mean Ranks	H	df	p value	Significant Dunn–Bonferroni pairwise comparisons
	Mean±SD											
Work–life balance and working hours	3.59±1.19	3.74±1.16	3.76±1.05	2.962	8	p=0.937	0.108	61.09; 65.35; 65.75	0.504	2	p=0.777	
Cooperation and communication with pet owners	3.48±1.11	3.88±1.09	3.65±1.01	6.901	8	p=0.547	0.165	58.79; 72.60; 62.43	3.382	2	p=0.184	
Cooperation and communication with colleagues	3.12±1.17	3.71±1.12	3.35±1.20	10.963	8	p=0.204	0.209	56.77; 74.93; 63.56	5.634	2	p=0.060	
Communication within the team	3.10±1.20	3.68±1.01	3.47±1.26	13.098	8	p=0.109	0.228	55.88; 72.65; 67.35	5.351	2	p=0.069	
Handling difficult medical cases	3.67±1.11	3.65±1.13	3.59±1.02	3.294	8	p=0.915	0.114	64.47; 64.22; 61.13	0.213	2	p=0.899	
Time management and scheduling tasks	3.26±0.98	3.50±1.19	3.68±0.84	29.702	8	*p<0.0001**	0.343	56.35; 65.56; 73.63	5.404	2	p=0.067	
Discussing payments with clients	3.50±1.23	3.32±1.36	3.71±0.94	11.561	8	p=0.111	0.214	63.20; 59.29; 68.22	1.086	2	p=0.581	
Pressure to generate income/revenue	2.69±1.38	3.26±1.42	2.91±1.36	8.515	8	p=0.385	0.184	58.03; 72.41; 63.93	3.490	2	p=0.175	
Time-consuming administrative tasks (e.g., phone calls, appointment coordination)	2.93±1.12	3.53±1.24	3.91±0.79	19.344	8	*p<0.05**	0.277	50.12; 69.66; 80.16	16.997	2	*p<0.0001**	23–34 vs. 35–44 years: p_adj = 0.031; 23–34 vs. ≥45 years: p_adj < 0.001
Professional development opportunities	3.43±1.01	3.56±1.19	2.88±1.01	11.492	8	p=0.175	0.214	66.59; 71.60; 50.13	7.202	2	*p<0.05**	35–44 vs. ≥45 years: p_adj = 0.035
Performing or deciding on euthanasia creates emotional or ethical difficulties	2.86±1.21	2.56±1.24	2.59±1.02	7.603	8	p=0.473	0.174	67.97; 58.57; 60.81	1.772	2	p=0.412	
Choosing the correct treatment	3.47±1.11	3.12±0.88	2.88±0.88	14.218	8	p=0.076	0.238	72.41; 60.40; 51.40	8.090	2	*p<0.05**	23–34 vs. ≥45 years: p_adj = 0.016
Balancing time between medical and administrative tasks	2.91±1.08	3.09±1.11	3.68±0.95	16.163	8	*p<0.05**	0.253	56.19; 59.66; 79.81	10.288	2	*p<0.01**	23–34 vs. ≥45 years: p_adj = 0.005
Communicating sensitive information to clients	3.14±1.10	3.12±0.95	3.09±1.19	8.362	8	p=0.399	0.182	64.39; 63.66; 61.82	0.115	2	p=0.944	
Making decisions under time pressure	3.43±1.22	3.76±1.23	3.35±1.18	21.303	8	*p<0.01**	0.291	61.59; 71.85; 58.40	2.758	2	p=0.252	
Balancing client expectations with medical quality	3.62±1.12	3.65±1.01	3.41±1.21	6.571	8	p=0.584	0.161	65.39; 65.10; 58.68	0.877	2	p=0.645	
Financial considerations in treatment decisions	3.59±1.30	3.88±0.88	3.68±1.01	10.412	8	p=0.237	0.203	61.80; 68.04; 61.85	0.775	2	p=0.679	
Delegating tasks to colleagues or assistants	2.72±1.23	3.12±1.01	2.71±1.00	8.211	8	p=0.413	0.181	60.22; 73.06; 59.53	3.431	2	p=0.180	
I am satisfied with my work–life balance.	2.62±1.23	2.85±1.31	2.94±1.10	4.073	8	p=0.850	0.127	59.18; 65.46; 68.91	1.756	2	p=0.416	
I feel comfortable in my team.	3.88±1.13	3.59±1.10	3.76±1.10	6.743	8	p=0.565	0.164	67.65; 56.62; 63.31	2.126	2	p=0.345	
I am able to talk to my colleagues when I am feeling overwhelmed.	3.86±1.10	3.47±1.31	3.68±1.17	4.832	8	p=0.775	0.138	67.75; 57.41; 62.34	1.896	2	p=0.387	
I feel comfortable talking to my supervisor during difficult situations.	3.72±1.24	3.18±1.45	3.29±1.22	15.890	8	*p<0.05**	0.251	70.74; 56.50; 58.15	4.504	2	p=0.105	
In my workplace, mental health and well-being are considered important topics.	2.84±1.25	3.00±1.30	3.29±1.38	8.362	8	p=0.399	0.182	59.14; 63.32; 71.12	2.415	2	p=0.299	
Overall, I am satisfied with my job.	3.60±0.99	3.82±0.87	3.94±1.01	7.349	8	p=0.499	0.171	57.71; 65.07; 71.81	3.722	2	p=0.156	

An asterisk (*) denotes a statistically significant association (p ≤ 0.05).

Supplementary Table S3. Differences in perceived occupational stressors and mental health outcomes by gender among veterinarians in Germany (n = 126).

Stressors and Statements (n=24)	Gender		Pearson's chi-square ($\chi 2$) test			Cramer's V value	Mann–Whitney U test			
	Female (n=101)	Male (n=25)	X-squared	df	p value		Mean Ranks	U	Z	p value
	Mean±SD									
Work–life balance and working hours	3.80±1.11	3.16±1.11	6.841	4	p=0.145	0.233	67.48; 47.42	860.5	-2.550	*p<0.05**
Cooperation and communication with pet owners	3.76±1.03	3.12±1.17	7.630	4	p=0.106	0.246	67.54, 47.16	854.0	-2.607	*p<0.01**
Cooperation and communication with colleagues	3.41±1.19	3.08±1.15	2.576	4	p=0.631	0.143	65.51; 55.38	1059.5	-1.280	p=0.200
Communication within the team	3.44±1.18	3.04±1.17	4.476	4	p=0.345	0.188	65.81, 54.16	1029.0	-1.472	p=0.141
Handling difficult medical cases	3.76±1.10	3.16±0.90	10.097	4	*p<0.05**	0.283	67.89; 45.76	819.0	-2.826	*p<0.01**
Time management and scheduling tasks	3.52±1.05	3.08±0.81	7.642	4	p=0.106	0.246	66.76; 50.34	933.5	-2.104	*p<0.05**
Discussing payments with clients	3.65±1.21	2.92±0.95	11.964	4	*p<0.05**	0.308	68.16; 44.66	791.5	-2.968	*p<0.01**
Pressure to generate income/revenue	3.01±0.46	2.48±1.01	8.763	4	p=0.067	0.264	66.01; 53.36	1009.0	-1.587	p=0.113
Time-consuming administrative tasks (e.g., phone calls, appointment coordination)	3.39±1.18	3.24±1.01	2.772	4	p=0.597	0.148	64.67; 58.76	1144.0	-0.751	p=0.453
Professional development opportunities	3.36±1.08	3.16±1.11	0.766	4	p=0.943	0.078	64.71; 58.62	1140.5	-0.777	p=0.437
Performing or deciding on euthanasia creates emotional or ethical difficulties	2.69±1.20	2.76±1.05	1.569	4	p=0.814	0.112	62.99; 65.58	1210.5	-0.328	p=0.743
Choosing the correct treatment	3.30±1.01	2.88±1.01	8.113	4	p=0.088	0.254	66.02; 53.32	1008.0	-1.624	p=0.104
Balancing time between medical and administrative tasks	3.17±1.12	3.16±0.99	3.455	4	p=0.485	0.166	63.30; 64.30	1242.5	-0.127	p=0.899
Communicating sensitive information to clients	3.18±1.06	2.88±1.13	9.424	4	p=0.051	0.273	64.93; 57.74	1118.5	-0.913	p=0.361
Making decisions under time pressure	3.59±1.20	3.12±1.20	3.889	4	p=0.421	0.176	66.36; 51.96	974.0	-1.817	p=0.069
Balancing client expectations with medical quality	3.77±1.01	2.76±1.17	20.023	4	*p<0.0001**	0.399	69.36; 39.82	670.5	-3.760	*p<0.0001**
Financial considerations in treatment decisions	3.89±1.00	2.88±1.24	23.483	4	*p<0.0001**	0.432	69.17; 40.58	689.5	-3.635	*p<0.0001**
Delegating tasks to colleagues or assistants	2.85±1.12	2.72±1.14	1.735	4	p=0.784	0.117	64.46; 59.64	1166.0	-0.611	p=0.541
I am satisfied with my work–life balance.	2.72±1.28	2.96±0.89	7.377	4	p=0.117	0.242	61.80; 70.36	1091.0	-1.080	p=0.280
I feel comfortable in my team.	3.75±1.14	3.84±0.99	2.621	4	p=0.623	0.144	63.26; 64.48	1238.0	-0.156	p=0.876
I am able to talk to my colleagues when I am feeling overwhelmed.	3.63±1.22	4.00±0.96	2.702	4	p=0.609	0.146	61.60; 71.18	1070.5	-1.217	p=0.223
I feel comfortable talking to my supervisor during difficult situations.	3.34±1.35	3.96±0.98	9.463	4	p=0.051	0.274	60.43; 75.90	952.5	-1.950	p=0.051
In my workplace, mental health and well-being are considered important topics.	2.90±1.32	3.44±1.19	4.676	4	p=0.322	0.193	60.65; 75.00	975.0	-1.799	p=0.072
Overall, I am satisfied with my job.	3.66±0.97	4.12±0.88	7.273	4	p=0.122	0.24	60.27; 76.54	936.5	-2.123	*p<0.05**

An asterisk (*) denotes a statistically significant association (p ≤ 0.05).

Supplementary Table S4. Differences in perceived occupational stressors and mental health outcomes by employment status among veterinarians in Germany (n = 126).

Stressors and Statements (n=24)	Employment status		Pearson's chi-square (χ2) test			Cramer's V value	Mann–Whitney U test			
	Employed (n=95)	Employer (n=31)	X-squared	df	p value		Mean Ranks	U	Z	p value
	Mean±SD									
Work–life balance and working hours	3.69±1.12	3.61±1.20	1.786	4	p=0.775	0.119	64.12; 61.61	1414.0	-0.344	p=0.731
Cooperation and communication with pet owners	3.61±1.11	3.71±1.01	4.474	4	p=0.346	0.188	63.12; 64.66	1436.5	-0.213	p=0.832
Cooperation and communication with colleagues	3.28±1.21	3.52±1.09	2.160	4	p=0.706	0.131	61.83; 68.61	1314.0	-0.926	p=0.355
Communication within the team	3.38±1.20	3.29±1.13	1.110	4	p=0.893	0.094	64.31; 61.02	1395.5	-0.450	p=0.653
Handling difficult medical cases	3.65±1.07	3.61±1.15	8.884	4	p=0.927	0.084	63.56; 63.31	1466.5	-0.035	p=0.972
Time management and scheduling tasks	3.45±1.00	3.39±1.09	1.679	4	p=0.795	0.115	63.76; 62.71	1448.0	-0.145	p=0.885
Discussing payments with clients	3.45±1.20	3.68±1.19	5.913	4	p=0.206	0.217	61.99; 68.11	1329.5	-0.834	p=0.404
Pressure to generate income/revenue	2.83±1.41	3.13±1.34	5.141	4	p=0.273	0.202	61.60; 69.32	1292.0	-1.046	p=0.295
Time-consuming administrative tasks (e.g., phone calls, appointment coordination)	3.23±1.16	3.74±1.03	5.443	4	p=0.245	0.208	59.53; 75.66	1095.5	-2.213	*p<0.05**
Professional development opportunities	3.41±1.01	3.03±1.28	7.019	4	p=0.135	0.236	66.25; 55.08	1211.5	-1.539	p=0.124
Performing or deciding on euthanasia creates emotional or ethical difficulties	2.76±1.16	2.55±1.18	2.164	4	p=0.706	0.131	65.09; 58.63	1321.5	-0.881	p=0.378
Choosing the correct treatment	3.34±1.03	2.84±0.90	6.567	4	p=0.161	0.228	67.58; 50.98	1084.5	-2.292	*p<0.05**
Balancing time between medical and administrative tasks	3.03±1.06	3.58±1.12	8.544	4	p=0.074	0.26	59.37; 76.15	1080.5	-2.313	*p<0.05**
Communicating sensitive information to clients	3.17±1.08	2.97±1.08	1.994	4	p=0.737	0.126	65.14; 58.48	1317.0	-0.913	p=0.361
Making decisions under time pressure	3.57±1.19	3.29±1.27	5.423	4	p=0.247	0.207	65.58; 57.11	1274.5	-1.155	p=0.248
Balancing client expectations with medical quality	3.65±1.06	3.32±1.25	4.301	4	p=0.367	0.185	65.88; 56.21	1246.5	-1.329	p=0.184
Financial considerations in treatment decisions	3.74±1.15	3.55±1.03	5.268	4	p=0.261	0.204	65.44; 57.56	1288.5	-1.081	p=0.280
Delegating tasks to colleagues or assistants	2.81±1.09	2.87±1.20	1.141	4	p=0.888	0.095	63.09; 64.76	1433.5	-0.229	p=0.819
I am satisfied with my work–life balance.	2.65±1.14	3.13±1.39	8.323	4	p=0.080	0.257	60.13; 73.84	1152.0	-1.870	p=0.062
I feel comfortable in my team.	3.72±1.16	3.94±0.96	4.196	4	p=0.380	0.182	62.17; 67.56	1346.5	-0.744	p=0.457
I am able to talk to my colleagues when I am feeling overwhelmed.	3.68±1.15	3.77±1.28	1.592	4	p=0.810	0.112	62.46; 66.68	1374.0	-0.578	p=0.563
I feel comfortable talking to my supervisor during difficult situations.	3.48±1.30	3.39±1.33	7.815	4	p=0.099	0.249	64.25; 61.21	1401.5	-0.414	p=0.679
In my workplace, mental health and well-being are considered important topics.	2.85±1.28	3.48±1.29	6.024	4	p=0.197	0.219	59.24; 76.56	1067.5	-2.346	*p<0.05**
Overall, I am satisfied with my job.	3.63±0.95	4.13±0.96	12.858	4	*p<0.05**	0.319	58.81; 77.89	1026.5	-2.690	*p<0.01**

An asterisk (*) denotes a statistically significant association (p ≤ 0.05).

Supplementary Table S5. Differences in perceived occupational stressors and mental health outcomes by weekly working hours among veterinarians in Germany (n = 126).

Stressors and Statements (n=24)	Weekly working hours		Pearson's chi-square (χ2) test			Cramer's V value	Mann–Whitney U test			
	≤ 40 hours (n=59)	> 40 hours (n=67)	X-squared	df	p value		Mean Ranks	U	Z	p value
	Mean±SD									
Work–life balance and working hours	3.53±1.12	3.81±1.15	9.010	4	p=0.061	0.267	58.44; 67.96	1678.0	-1.513	p=0.130
Cooperation and communication with pet owners	3.61±1.09	3.66±1.10	3.738	4	p=0.443	0.172	62.47; 64.40	1916.0	-0.309	p=0.758
Cooperation and communication with colleagues	3.31±1.24	3.37±1.14	7.412	4	p=0.116	0.243	62.75; 64.16	1932.0	-0.224	p=0.823
Communication within the team	3.32±1.17	3.39±1.21	2.399	4	p=0.663	0.138	62.43; 64.44	1913.5	-0.317	p=0.751
Handling difficult medical cases	3.53±1.10	3.75±1.06	2.421	4	p=0.659	0.139	59.91; 66.66	1764.5	-1.080	p=0.280
Time management and scheduling tasks	3.36±1.03	3.51±1.01	1.189	4	p=0.880	0.097	60.50; 66.14	1799.5	-0.904	p=0.366
Discussing payments with clients	3.44±1.16	3.57±1.23	6.446	4	p=0.168	0.226	61.25; 65.49	1843.5	-0.670	p=0.503
Pressure to generate income/revenue	2.88±1.34	2.93±1.45	2.129	4	p=0.712	0.13	63.03; 63.91	1949.0	-0.138	p=0.891
Time-consuming administrative tasks (e.g., phone calls, appointment coordination)	3.17±1.12	3.52±1.16	4.208	4	p=0.379	0.183	57.36; 68.91	1614.0	-1.836	p=0.066
Professional development opportunities	3.36±1.01	3.28±1.15	1.923	4	p=0.750	0.124	64.28; 62.81	1930.5	-0.234	p=0.815
Performing or deciding on euthanasia creates emotional or ethical difficulties	2.95±1.27	2.49±1.04	9.029	4	p=0.060	0.268	70.74; 57.13	1549.5	-2.150	*p<0.05**
Choosing the correct treatment	3.29±1.07	3.15±0.97	2.220	4	p=0.695	0.133	65.91; 61.38	1834.5	-0.724	p=0.469
Balancing time between medical and administrative tasks	3.00±1.00	3.31±1.16	5.169	4	p=0.270	0.203	58.23; 68.14	1665.5	-1.584	p=0.113
Communicating sensitive information to clients	3.20±0.98	3.04±1.16	4.396	4	p=0.355	0.187	65.86; 61.43	1837.5	-0.705	p=0.481
Making decisions under time pressure	3.63±1.19	3.39±1.23	1.241	4	p=0.871	0.099	67.20; 60.24	1758.0	-1.100	p=0.271
Balancing client expectations with medical quality	3.58±1.12	3.57±1.12	0.775	4	p=0.942	0.078	63.70; 63.32	1964.5	-0.061	p=0.951
Financial considerations in treatment decisions	3.76±1.15	3.63±1.10	4.072	4	p=0.396	0.18	66.07; 61.25	1826.0	-0.763	p=0.445
Delegating tasks to colleagues or assistants	2.95±1.15	2.72±1.08	2.040	4	p=0.728	0.127	67.47; 60.00	1742.0	-1.187	p=0.235
I am satisfied with my work–life balance.	3.02±1.18	2.55±1.21	8.501	4	p=0.075	0.26	70.53; 57.31	1561.5	-2.090	*p<0.05**
I feel comfortable in my team.	3.68±1.14	3.85±1.09	1.088	4	p=0.896	0.093	60.57; 66.08	1803.5	-0.882	p=0.378
I am able to talk to my colleagues when I am feeling overwhelmed.	3.75±1.17	3.67±1.20	0.875	4	p=0.928	0.083	64.56; 62.57	1914.0	-0.317	p=0.751
I feel comfortable talking to my supervisor during difficult situations.	3.56±1.28	3.37±1.34	2.463	4	p=0.651	0.14	66.22; 61.1	1816.0	-0.807	p=0.420
In my workplace, mental health and well-being are considered important topics.	3.02±1.28	3.00±1.34	4.935	4	p=0.294	0.198	63.49; 63.51	1976.0	-0.003	p=0.998
Overall, I am satisfied with my job.	3.76±0.94	3.75±1.01	2.945	4	p=0.567	0.153	63.81; 63.23	1958.5	-0.094	p=0.925

An asterisk (*) denotes a statistically significant association (p ≤ 0.05).

Supplementary Table S6. Differences in perceived occupational stressors and mental health outcomes by professional experience among veterinarians in Germany (n = 126).

Stressors and Statements (n=24)	Professional experience				Pearson's chi-square (χ2) test			Cramer's V value	Kruskal–Wallis test				
	0-3 years (n=44)	4-10 years (n=35)	11-20 years (n=18)	> 20 years (n=29)	X-squared	df	p value		Mean Ranks	H	df	p value	Significant Dunn–Bonferroni pairwise comparisons
	Mean±SD												
Work–life balance and working hours	3.59±1.25	3.66±1.06	3.83±1.20	3.72±1.07	6.791	12	p=0.817	0.134	61.64; 62.06; 69.08; 64.60	0.663	3	p=0.882	
Cooperation and communication with pet owners	3.48±1.19	3.60±0.98	4.11±1.08	3.62±1.01	14.836	12	p=0.251	0.198	59.75; 60.67; 80.75; 61.90	5.168	3	p=0.160	
Cooperation and communication with colleagues	3.02±1.21	3.51±1.01	3.72±1.23	3.38±1.24	11.433	12	p=0.492	0.174	54.27; 68.16; 75.33; 64.53	5.625	3	p=0.131	
Communication within the team	3.09±1.20	3.37±1.11	4.11±0.90	3.28±1.25	12.704	12	p=0.391	0.183	55.06; 63.47; 86.25; 61.40	9.710	3	*p<0.05**	0–3 years vs. 11–20 years: p_adj = 0.012
Handling difficult medical cases	3.61±1.24	3.80±0.93	3.61±1.09	3.52±1.02	8.639	12	p=0.733	0.151	63.94; 67.37; 62.14; 59.00	0.939	3	p=0.816	
Time management and scheduling tasks	3.32±1.03	3.31±0.96	3.56±1.25	3.69±0.89	28.507	12	*p<0.01**	0.275	58.95; 57.56; 68.42; 74.52	4.998	3	p=0.172	
Discussing payments with clients	3.34±1.18	3.40±1.40	3.83±1.15	3.69±0.97	13.107	12	p=0.361	0.186	58.39; 61.20; 73.50; 67.83	2.927	3	p=0.403	
Pressure to generate income/revenue	2.61±1.35	2.94±1.45	3.39±1.29	3.00±1.41	7.552	12	p=0.819	0.141	55.99; 64.46; 76.06; 65.95	4.339	3	p=0.227	
Time-consuming administrative tasks (e.g., phone calls, appointment coordination)	2.84±1.08	3.26±1.25	3.78±1.06	4.00±0.76	26.363	12	*p<0.01**	0.264	47.13; 60.81; 77.44; 82.93	21.335	3	*p<0.0001**	0–3 years vs. 11–20 years: p_adj = 0.013; 0–3 years vs. >20 years: p_adj < 0.001
Professional development opportunities	3.52±1.02	3.54±1.07	3.17±1.30	2.83±0.93	18.355	12	p=0.105	0.22	69.72; 70.03; 60.47; 48.07	8.341	3	*p<0.05**	No significant pairwise difference after Bonferroni correction
Performing or deciding on euthanasia creates emotional or ethical difficulties	3.00±1.18	2.43±1.07	2.44±1.50	2.76±0.95	24.572	12	*p<0.05**	0.255	72.61; 54.70; 54.08; 66.14	6.493	3	p=0.090	
Choosing the correct treatment	3.59±1.04	3.14±1.00	2.89±0.90	2.93±0.92	15.193	12	p=0.231	0.2	76.74; 60.79; 52.69; 53.40	10.631	3	*p<0.05**	0–3 years vs. >20 years: p_adj = 0.032
Balancing time between medical and administrative tasks	2.84±1.12	3.03±1.04	3.22±1.06	3.79±0.90	20.337	12	p=0.061	0.232	54.16; 58.74; 63.17; 83.17	13.324	3	*p<0.01**	0–3 years vs. >20 years: p_adj = 0.003; 4–10 years vs. >20 years: p_adj = 0.028
Communicating sensitive information to clients	3.07±1.15	3.20±0.96	3.06±0.87	3.14±1.25	14.372	12	p=0.278	0.195	62.17; 66.17; 61.28; 63.55	0.351	3	p=0.950	
Making decisions under time pressure	3.41±1.15	3.66±1.21	3.61±1.46	3.38±1.18	20.631	12	p=0.056	0.234	60.51; 68.21; 68.56; 59.21	1.722	3	p=0.632	
Balancing client expectations with medical quality	3.52±1.19	3.77±1.03	3.67±0.91	3.34±1.20	7.842	12	p=0.797	0.144	62.59; 69.61; 65.03; 56.55	2.252	3	p=0.522	
Financial considerations in treatment decisions	3.39±1.32	3.89±0.99	3.89±0.90	3.79±1.01	13.305	12	p=0.347	0.188	55.78; 68.74; 68.28; 65.91	3.355	3	p=0.340	
Delegating tasks to colleagues or assistants	2.75±1.22	3.00±1.16	2.78±0.88	2.76±1.06	8.011	12	p=0.784	0.146	61.06; 68.99; 61.89; 61.59	1.182	3	p=0.757	
I am satisfied with my work–life balance.	2.66±1.22	2.46±1.20	3.22±1.26	3.03±1.12	12.825	12	p=0.382	0.184	60.77; 54.13; 75.53; 71.48	6.247	3	p=0.100	
I feel comfortable in my team.	3.89±1.06	3.69±1.05	3.67±1.33	3.76±1.15	9.058	12	p=0.698	0.155	66.95; 59.73; 61.97; 63.76	0.870	3	p=0.833	

I am able to talk to my colleagues when I am feeling overwhelmed.	3.82±1.15	3.77±1.11	3.39±1.42	3.66±1.17	6.933	12	p=0.862	0.135	66.69; 64.83; 56.08; 61.66	1.289	3	p=0.732	
I feel comfortable talking to my supervisor during difficult situations.	3.68±1.25	3.51±1.27	3.22±1.56	3.21±1.26	14.632	12	p=0.262	0.197	69.48; 64.53; 58.83; 56.09	2.850	3	p=0.415	
In my workplace, mental health and well-being are considered important topics.	2.93±1.27	2.89±1.21	2.72±1.53	3.45±1.30	11.329	12	p=0.501	0.173	61.44; 60.13; 56.08; 75.29	4.399	3	p=0.221	
Overall, I am satisfied with my job.	3.64±0.92	3.63±1.00	3.89±1.08	4.00±0.93	9.954	12	p=0.620	0.162	58.52; 58.41; 70.36; 72.93	4.609	3	p=0.203	

An asterisk (*) denotes a statistically significant association ($p \leq 0.05$).

Supplementary Table S7. Differences in perceived occupational stressors and mental health outcomes by current mental health status among veterinarians in Germany (n = 126).

Stressors and Statements (n=24)	Current mental health status			Pearson's chi-square ($\chi 2$) test			Cramer's V value	Kruskal–Wallis test				
	Very Good and Good (n=67)	Satisfactory (n=45)	Poor and Very Poor (n=14)	X-squared	df	p value		Mean Ranks	H	df	p value	Significant Dunn–Bonferroni pairwise comparisons
	Mean±SD											
Work–life balance and working hours	3.58±1.10	3.80±1.18	3.71±1.20	9.806	8	p=0.279	0.197	60.57; 67.53; 64.54	1.065	2	p=0.587	
Cooperation and communication with pet owners	3.48±1.16	3.84±0.95	3.71±1.07	9.987	8	p=0.266	0.199	59.30; 69.29; 65.00	2.223	2	p=0.329	
Cooperation and communication with colleagues	3.28±1.15	3.44±1.27	3.29±1.07	5.133	8	p=0.743	0.143	61.72; 66.91; 61.07	0.653	2	p=0.721	
Communication within the team	3.22±1.22	3.49±1.18	3.57±1.02	7.193	8	p=0.516	0.169	59.78; 67.32; 69.00	1.598	2	p=0.450	
Handling difficult medical cases	3.55±1.06	3.71±1.12	3.86±1.10	3.587	8	p=0.892	0.119	60.18; 65.94; 71.54	1.555	2	p=0.459	
Time management and scheduling tasks	3.27±1.04	3.56±0.94	3.86±1.03	7.626	8	p=0.471	0.174	57.57; 67.70; 78.36	5.109	2	p=0.078	
Discussing payments with clients	3.36±1.11	3.87±1.24	3.07±1.27	13.906	8	p=0.084	0.235	58.43; 74.90; 51.14	7.726	2	*p<0.05**	Very good/good vs. Satisfactory: p_adj = 0.048
Pressure to generate income/revenue	2.48±1.25	3.24±1.38	3.86±1.41	22.199	8	*p<0.01**	0.297	52.79; 72.18; 86.86	14.691	2	*p<0.001**	Very good/good vs. Satisfactory: p_adj = 0.014; Very good/good vs. Poor/very poor: p_adj = 0.003
Time-consuming administrative tasks (e.g., phone calls, appointment coordination)	3.25±1.13	3.40±1.18	3.71±1.14	8.010	8	p=0.432	0.178	60.22; 64.99; 74.39	1.997	2	p=0.369	
Professional development opportunities	3.31±1.13	3.16±0.95	3.86±1.17	12.010	8	p=0.151	0.218	63.80; 58.08; 79.50	3.993	2	p=0.136	
Performing or deciding on euthanasia creates emotional or ethical difficulties	2.46±1.11	3.04±1.21	2.79±1.12	10.920	8	p=0.206	0.208	55.94; 73.72, 66.82	6.908	2	*p<0.05**	Very good/good vs. Satisfactory: p_adj = 0.028
Choosing the correct treatment	2.97±1.00	3.49±0.97	3.50±1.02	14.333	8	p=0.073	0.238	54.93; 73.66; 71.86	8.596	2	*p<0.05**	Very good/good vs. Satisfactory: p_adj = 0.017
Balancing time between medical and administrative tasks	3.15±1.03	3.13±1.20	3.36±1.08	5.346	8	p=0.720	0.146	63.05; 62.94; 67.43	0.198	2	p=0.906	
Communicating sensitive information to clients	3.00±1.06	3.27±1.12	3.21±1.05	10.238	8	p=0.249	0.202	60.43; 67.54; 65.21	1.137	2	p=0.566	
Making decisions under time pressure	3.24±1.21	3.73±1.12	4.00±1.30	10.868	8	p=0.209	0.208	55.63; 70.19; 79.68	7.817	2	*p<0.05**	No significant pairwise difference after Bonferroni correction
Balancing client expectations with medical quality	3.30±1.07	3.76±1.07	4.29±1.07	17.743	8	*p<0.05**	0.265	54.40; 69.10; 89.04	13.000	2	*p<0.01**	Very good/good vs. Poor/very poor: p_adj = 0.002

Financial considerations in treatment decisions	3.42±1.14	4.04±1.00	3.86±1.10	16.973	8	*p<0.05**	0.26	54.92; 74.91; 67.89	8.921	2	*p<0.05**	Very good/good vs. Satisfactory: p_adj = 0.010
Delegating tasks to colleagues or assistants	2.73±1.11	2.91±1.18	3.00±0.96	4.662	8	p=0.793	0.136	60.26; 66.28; 70.07	1.331	2	p=0.514	
I am satisfied with my work–life balance.	3.31±1.06	2.38±1.09	1.43±0.65	46.501	8	*p<0.0001**	0.43	79.90; 51.53; 23.50	37.270	2	*p<0.0001**	Very good/good vs. Satisfactory: p_adj < 0.001; Very good/good vs. Poor/very poor: p_adj < 0.001; Satisfactory vs. Poor/very poor: p_adj = 0.029
I feel comfortable in my team.	4.00±1.14	3.71±0.90	2.86±1.17	22.374	8	*p<0.01**	0.298	72.17; 59.12; 36.07	13.394	2	*p<0.0001**	Very good/good vs. Poor/very poor: p_adj = 0.001
I am able to talk to my colleagues when I am feeling overwhelmed.	4.09±1.08	3.42±1.16	2.79±0.98	25.211	8	*p<0.001**	0.316	75.60; 54.31; 35.14	20.031	2	*p<0.0001**	Very good/good vs. Satisfactory: p_adj = 0.005; Very good/good vs. Poor/very poor: p_adj < 0.001
I feel comfortable talking to my supervisor during difficult situations.	3.84±1.27	3.33±1.17	2.07±0.83	39.210	8	*p<0.0001**	0.394	74.71; 58.51; 25.89	23.259	2	*p<0.0001**	Very good/good vs. Poor/very poor: p_adj < 0.001; Satisfactory vs. Poor/very poor: p_adj = 0.008
In my workplace, mental health and well-being are considered important topics.	3.42±1.25	2.71±1.24	2.00±1.04	22.907	8	*p<0.01**	0.301	74.90; 55.08; 36.00	17.646	2	*p<0.0001**	Very good/good vs. Satisfactory: p_adj = 0.012; Very good/good vs. Poor/very poor: p_adj < 0.001
Overall, I am satisfied with my job.	4.16±0.81	3.53±0.76	2.50±1.02	55.456	8	*p<0.0001**	0.469	78.71; 52.76; 25.25	35.003	2	*p<0.0001**	Very good/good vs. Satisfactory: p_adj < 0.001; Very good/good vs. Poor/very poor: p_adj < 0.001; Satisfactory vs. Poor/very poor: p_adj = 0.026

An asterisk (*) denotes a statistically significant association (p ≤ 0.05).

Supplementary Table S8. Differences between perceived occupational stressors and mental health outcomes by annual leave entitlement among veterinarians in Germany (n = 126).

Stressors and Statements (n=24)	Holidays		Pearson's chi-square (χ2) test			Cramer's V value	Mann–Whitney U test			
	≤ 28 days (n=64)	> 28 days (n=62)	X-squared	df	p value		Mean Ranks	U	Z	p value
	Mean±SD									
Work–life balance and working hours	3.83±1.11	3.52±1.16	5.159	4	p=0.271	0.202	68.02; 58.83	1694.5	-1.465	p=0.143
Cooperation and communication with pet owners	3.69±1.13	3.58±1.05	4.335	4	p=0.363	0.185	65.36; 61.58	1865.0	-0.606	p=0.545
Cooperation and communication with colleagues	3.27±1.17	3.42±1.20	3.497	4	p=0.478	0.167	60.94; 66.15	1820.0	-0.825	p=0.409
Communication within the team	3.36±1.17	3.35±1.20	1.994	4	p=0.737	0.126	63.54; 63.46	1981.5	-0.013	p=0.990
Handling difficult medical cases	3.81±1.05	3.47±1.10	5.180	4	p=0.269	0.203	68.83; 58.00	1643.0	-1.733	p=0.083
Time management and scheduling tasks	3.59±1.02	3.27±1.00	3.864	4	p=0.425	0.175	69.13; 57.69	1623.5	-1.839	p=0.066
Discussing payments with clients	3.45±1.23	3.56±1.17	2.747	4	p=0.601	0.148	62.09; 64.95	1894.0	-0.452	p=0.651
Pressure to generate income/revenue	3.06±1.50	2.74±1.27	6.546	4	p=0.162	0.228	67.16; 59.72	1749.5	-1.171	p=0.242
Time-consuming administrative tasks (e.g., phone calls, appointment coordination)	3.31±1.27	3.40±1.02	6.293	4	p=0.178	0.223	63.34; 63.66	1974.0	-0.051	p=0.960
Professional development opportunities	3.23±1.15	3.40±1.02	4.479	4	p=0.345	0.189	61.33; 65.74	1845.0	-0.706	p=0.480
Performing or deciding on euthanasia creates emotional or ethical difficulties	2.91±1.24	2.50±1.05	6.774	4	p=0.148	0.232	69.53; 57.27	1598.0	-1.940	p=0.052
Choosing the correct treatment	3.31±0.96	3.11±1.07	5.120	4	p=0.275	0.202	66.24; 60.67	1808.5	-0.893	p=0.372
Balancing time between medical and administrative tasks	3.14±1.13	3.19±1.07	1.316	4	p=0.859	0.102	63.08; 63.94	1957.0	-0.137	p=0.891
Communicating sensitive information to clients	3.14±1.05	3.10±1.11	0.654	4	p=0.957	0.072	64.01; 62.98	1951.5	-0.164	p=0.869
Making decisions under time pressure	3.66±1.14	3.34±1.27	10.312	4	*p<0.05**	0.286	67.92; 58.94	1701.0	-1.422	p=0.155
Balancing client expectations with medical quality	3.59±1.07	3.55±1.17	4.781	4	p=0.311	0.195	63.87; 63.12	1960.5	-0.119	p=0.905
Financial considerations in treatment decisions	3.72±1.12	3.66±1.13	2.874	4	p=0.579	0.151	64.52; 62.44	1918.5	-0.331	p=0.740
Delegating tasks to colleagues or assistants	2.94±1.07	2.71±1.17	2.320	4	p=0.677	0.136	67.16; 59.73	1750.0	-1.183	p=0.237
I am satisfied with my work–life balance.	2.69±1.27	2.85±1.16	4.062	4	p=0.398	0.18	61.27; 65.80	1841.5	-0.716	p=0.474
I feel comfortable in my team.	3.61±1.20	3.94±0.99	3.515	4	p=0.476	0.167	59.16; 67.98	1706.0	-1.414	p=0.157
I am able to talk to my colleagues when I am feeling overwhelmed.	3.63±1.23	3.79±1.13	2.237	4	p=0.692	0.133	61.31; 65.76	1844.0	-0.708	p=0.479
I feel comfortable talking to my supervisor during difficult situations.	3.22±1.37	3.71±1.19	11.401	4	*p<0.05**	0.301	57.17; 70.03	1579.0	-2.032	*p<0.05**
In my workplace, mental health and well-being are considered important topics.	2.83±1.41	3.19±1.17	6.793	4	p=0.147	0.232	58.69; 68.47	1676.0	-1.537	p=0.124
Overall, I am satisfied with my job.	3.61±1.08	3.90±0.82	6.852	4	p=0.144	0.233	59.05; 68.10	1699.0	-1.481	p=0.139

An asterisk (*) denotes a statistically significant association (p ≤ 0.05).
